# COPI-regulated mitochondria-ER contact site formation maintains axonal integrity

**DOI:** 10.1016/j.celrep.2023.112883

**Published:** 2023-07-26

**Authors:** Daniel C. Maddison, Bilal Malik, Leonardo Amadio, Dana M. Bis-Brewer, Stephan Züchner, Owen M. Peters, Gaynor A. Smith

**Affiliations:** 1 UK Dementia Research Institute, School of Medicine, Cardiff University, Cardiff CF24 4HQ, UK; 2 UK Dementia Research Institute, School of Biosciences, Cardiff University, Cardiff CF24 4HQ, UK; 3 John P. Hussman Institute for Human Genomics, University of Miami, Miami, FL, USA; 4 Dr. John T. Macdonald Foundation Department of Human Genetics, University of Miami, Miami, FL, USA

**Keywords:** Neuronal cell biology, Mitochondria, Endoplasmic reticulum, Golgi, COPI, Vesicle trafficking, Axon transport, Calcium homeostasis

## Abstract

Coat protein complex I (COPI) is best known for its role in Golgi-endoplasmic reticulum (ER) trafficking, responsible for the retrograde transport of ER-resident proteins. The ER is crucial to neuronal function, regulating Ca^2+^ homeostasis and the distribution and function of other organelles such as endosomes, peroxisomes, and mitochondria via functional contact sites. Here we demonstrate that disruption of COPI results in mitochondrial dysfunction in *Drosophila* axons and human cells. The ER network is also disrupted, and the neurons undergo rapid degeneration. We demonstrate that mitochondria-ER contact sites (MERCS) are decreased in COPI-deficient axons, leading to Ca^2+^ dysregulation, heightened mitophagy, and a decrease in respiratory capacity. Reintroducing MERCS is sufficient to rescue not only mitochondrial distribution and Ca^2+^ uptake but also ER morphology, dramatically delaying neurodegeneration. This work demonstrates an important role for COPI-mediated trafficking in MERC formation, which is an essential process for maintaining axonal integrity.

## Highlights

• Loss of COPI subunits results in mitochondrial dysfunction in neurons
• COPI dysfunction disrupts axonal ER and Ca^2+^ homeostasis, causing rapid neurodegeneration
• Mitochondrial-ER contact sites are decreased when Golgi-ER trafficking is disrupted
• Boosting MERCS rescues axonal ER and degeneration independent of mitochondrial morphology

## Graphical abstract

**Figure:**
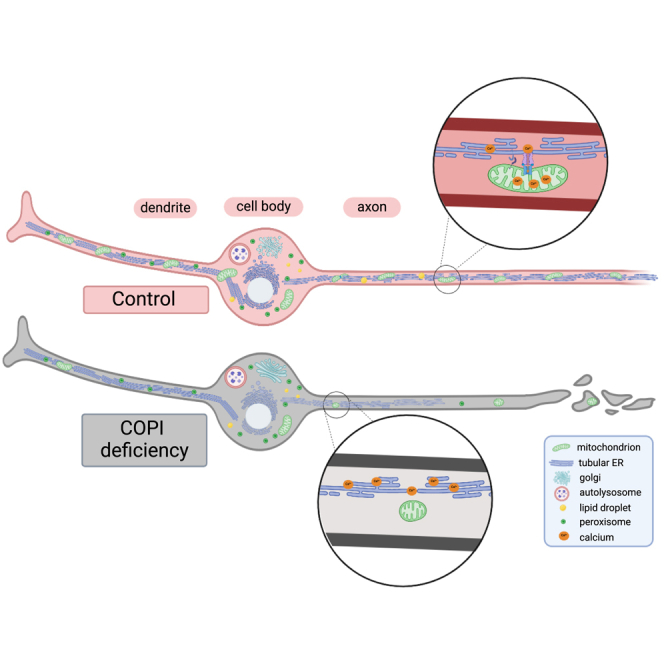

## Teaser

Coat protein complex I (COPI) is understood for its role in Golgi-to-ER trafficking in cells. Maddison et al. show that COPI is also essential for the maintenance of adult neurons including their long axonal projections, functioning to promote contact sites between the ER and the mitochondrial network.

## Introduction

Coat protein complex I (COPI) has a well-described role in the bidirectional transport of lipids and proteins between the *cis*-Golgi and endoplasmic reticulum (ER). The complex consists of seven core subunits, αCOP, βCOP, β′COP, γCOP, δCOP, εCOP, and ζCOP, which form a coatomer coat around membrane-bound vesicles, facilitating their trafficking between Golgi and ER.^1^ Although the best-characterized COPI function is the retrograde transport of cargo from Golgi to ER,^2^ it has also been shown to play a role in the sorting of cargo for anterograde ER to Golgi transport at ER exit sites^3^^,^^4^^,^^5^ and inter-Golgi trafficking.^6^ Formation of COPI coatomer is initiated via interaction with the small guanosine triphosphatase (GTPase) ADP-ribosylation factor isoforms (ARF1, ARF4, and ARF5),^7^^,^^8^ which recruit the heptameric complex,^9^ each isoform defining a specific COPI function in bidirectional Golgi-ER sorting.^6^ The ER has been found to stretch continuously throughout neurons including in long axons^10^^,^^11^^,^^12^; however, the significance of Golgi-ER transport in this cell type is poorly defined. It remains unclear how COPI-mediated transport may impact on the integrity of the ER and ultimately the survival of adult neurons *in vivo*, including axon stretches most susceptible to neurodegeneration.

Disruption of Golgi-ER trafficking via inhibition of ARF1 or its effector protein, Golgi-specific brefeldin A-resistance guanine nucleotide exchange factor, has also been shown to affect mitochondrial morphology in multiple models.^13^^,^^14^^,^^15^ However, since ARF1 regulates multiple modes of Golgi to ER transport, such as phosphatidylinositol 4-phosphate (PI(4)P) vesicles^16^ and Rab6-mediated Golgi tubules^17^ as well as COPI vesicles, the specific mechanism and role for COPI in the control of mitochondrial function is unclear.^13^ Mitochondria are dynamic organelles that undergo fission and fusion events to maintain the health of their overall population within a cell.^18^ Mitochondrial dynamics are intrinsically linked to quality control mechanisms such as mitophagy^19^^,^^20^ and mitochondrial-derived vesicle formation,^21^ as well as contacts with other cellular structures such as microtubules and the ER.^18^^,^^22^

Given the role of COPI in retrograde cargo delivery to the ER, we hypothesized that this specialized form of intracellular transport may be important for establishing mitochondria-ER contact sites (MERCS). MERCS serve important cellular functions, including exchange of lipids and calcium (Ca^2+^).^23^ Proteins involved in mitochondrial dynamics, such as mitofusin 2 (MFN2) and mitochondrial rho GTPase (MIRO1/2), localize to and regulate MERCS.^24^^,^^25^^,^^26^ Functional interactions between several other mitochondrial proteins, such as protein tyrosine phosphatase-interacting protein 51 (PTPIP51),^27^ voltage-dependent anion channel (VDAC),^28^ and translocase of outer mitochondrial membrane (TOMM40),^29^ with respective ER proteins such as vesicle-associated membrane protein B (VAPB), inositol 1,4,5-triphosphate receptor (IP3R), and B cell receptor-associated protein 31 (BAP31), have also been identified.^23^ It is well understood that the ER plays a key role in the regulation of the mitochondrial network in neurons.^30^ However, it is unknown whether and how Golgi-ER transport can facilitate this, particularly in long axon stretches at distances far away from the cell soma, in the absence of a conventional Golgi network.^31^

In this study, we report that loss of COPI α-subunit (αCOP) in neurons causes mitochondrial fragmentation, disruption of the ER, and a decrease in axonal MERCS, leading to rapid neurodegeneration. Overexpression of Miro, Marf (a synthetic ER-mitochondrial tether), or the fly VAPB ortholog (Vap33) rescued MERCS, mitochondrial Ca^2+^, and axonal ER, and dramatically delayed the onset of neurodegeneration, independent from changes in mitochondrial morphology. This suggests that Golgi-ER trafficking is required for MERC formation and ER maintenance in axons, which is fundamental for neuronal survival *in vivo*.

## Results

### A null mutation in *αCOP* results in abnormal mitochondria in neurons and rapid-onset neurodegeneration

**Figure 1:**
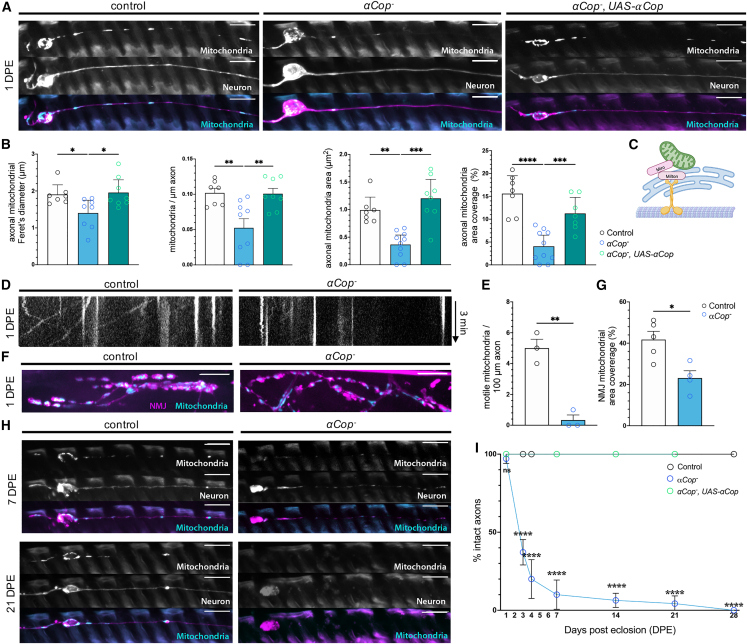
*αCOP* mutation disrupts mitochondrial morphology, transport, and distribution in *Drosophila* neurons, leading to neurodegeneration (A) Mitochondria labeled by mito::GFP and neuronal membrane labeled by myr::tdTomato in *Drosophila* glutamatergic sensory wing neurons of control, *αCOP*^−^ mutant, and *αCOP*^−^, *UAS-αCOP* rescue clones at 1 day post eclosion (DPE). (B) Median mitochondrial Feret’s diameter per axon, number of mitochondria per μM axon, median mitochondrial area per axon, and mitochondrial area coverage per axon values are significantly decreased by αCOP deficiency. Each data point represents mean value for an individual fly, calculated across 5–10 clones from both wings. (C) The number and size of mitochondria depend on tethering to the microtubules via adapter proteins and to the ER. (D) Kymographs representing mito::GFP-labeled mitochondrial motility over 3 min in control and *αCOP*^−^ mutant neurons reveal significant deficits in mitochondrial transport. (E) Mean number of motile mitochondria during 3 min in control and *αCOP*^−^ neurons is significantly decreased. Each data point represents mean value for an individual fly, calculated across both wings. (F) Mito::GFP-labeled mitochondria in synaptic boutons of control and *αCOP*^−^ mutant leg motor neuromuscular junctions (NMJs). (G) Area coverage of mitochondria in control and *αCOP*^−^ NMJs are significantly reduced. Each data point represents data from a single leg from a single fly. (H) *Drosophila* glutamatergic sensory wing neurons of control, *αCOP*^−^ mutant, and *αCOP*^−^, *UAS-αCOP* rescue clones at 7 and 21 DPE. (I) Percentage of intact axons in control, *αCOP*^−^ mutant, and *αCOP*^−^, rescued by *UAS-αCOP* neurons. Data points represent mean percentage of intact neurons across >6 individual flies, with 5–10 clones assessed per fly from both wings. One-way and two-way ANOVAs with false discovery rate (FDR) correction or Student’s t test were used. Graphs are presented as mean ± SD, and significance is annotated as: ns, not significant; ^∗^p < 0.05, ^∗∗^p < 0.01, ^∗∗∗^p < 0.001, ^∗∗∗^p < 0.0001. All scale bars, 10 μm.

Through unbiased forward genetic screening using *Drosophila melanogaster* neurons, we uncovered mutant *l*(*3*)*2750*, which caused a marked reduction in the number and size of mitochondria in axons (Figures 1A and 1B). We have previously established this screen in search of mutants with altered mitochondrial size or distribution in the axons of glutamatergic sensory neurons of the adult L1 wing vein.^32^^,^^33^ The chemical mutagen ethyl methanesulfonate was used to induce DNA mutations at random genomic locations, and the MARCM (mosaic analysis with a repressible cell marker) system was used to induce homozygous mutant clones in F_1_ progeny so that neurons and mitochondria they contain could be visualized using genetically encoded markers at single-cell resolution.^32^^,^^34^^,^^35^ Importantly this clonal approach allows for in-depth characterization of genes that would otherwise be lethal if knocked out in the whole animal or nervous system. The *l*(*3*)*2750* mutant was subsequently named *αCOP*^−^ after whole-genome sequencing confirmed a 28 bp deletion and the introduction of a premature stop codon in the *αCOP* gene as the phenotype-causing lesion (Figure S1A). The mutation is homozygous lethal, causing early larval lethality, and fails to complement a deficiency strain in which *αCOP* has been deleted, and thus can be considered a null allele (Figure S1B).

*αCOP* deletion caused a significant decrease in the number, length, area, and axon coverage of mitochondria in axons compared to control neurons at 1 day post eclosion (DPE), phenotypes which were rescued by re-expressing the gene in the clonal knockout neurons^36^ (Figures 1A and 1B). The distribution of mitochondria in axons is dependent on tethering to microtubule motor proteins kinesin and dynein via the adapters Miro and Milton to facilitate anterograde and retrograde transport^37^^,^^38^^,^^39^ (Figure 1C). We thus next investigated mitochondrial trafficking in *αCOP*-deficient axons in living flies.^40^ The proportion of motile mitochondria per axon was significantly decreased in *αCOP*^−^ wing neurons at 1 DPE (Figures 1D and 1E). At the neuromuscular junction of leg motor neurons, mitochondrial area coverage was significantly reduced (Figures 1F and 1G), suggesting that a decrease in anterograde mitochondrial trafficking from the cell body is preventing mitochondria from reaching the nerve terminals. Wing neurons underwent rapid degeneration, and by 7 DPE only <10% of cells retained intact axons (Figures 1H and 1I). By 28 DPE, no intact axons remained in *αCOP*^−^ neurons. The neurodegenerative phenotype was also fully rescued by overexpression of αCOP (Figure 1I). αCOP can therefore be considered important for mitochondrial dynamics, mitochondrial transport, and the survival of adult neurons.

Since αCOP is a subunit of the heptameric COPI complex, we next investigated whether the observed phenotypes were specific to αCOP or a consequence of general COPI disruption. We produced *γCOP*^10^ homozygous mutant clones in wing neurons and observed similar mitochondrial and neurodegenerative phenotypes (Figures S2A, S2C, and S2D). Rab6 regulates both COPI-dependent^41^ and -independent^42^ retrograde Golgi-ER transport of specific cargo. Rab6::GFP appeared to mislocalize in *αCOP* mutant neurons and was diffuse in the Golgi-rich cell-body compartment (Figure S2B), indicating that without the COPI, Rab6 function is impaired. We therefore produced *Rab6*^−^ mutant neurons, to assess whether the mitochondrial phenotypes observed could be caused downstream of COPI by Rab6-dependent pathways. No significant differences in mitochondria size or number were observed in *Rab6*^−^ mutant neurons (Figures S2A and S2C), which also retained intact axons beyond 21 DPE (Figure S2D). The observed mitochondrial deficits are therefore caused via COPI-dependent but Rab6-independent mechanisms. We also tested two mediators of anterograde ER to Golgi transport, the GTPases Rab1 and Sar1. *Rab1* mutant clones did not reproduce mitochondrial or neurodegenerative phenotypes. However, loss of *Sar1*, which controls coat assembly of COPII vesicles, caused a dramatic reduction in mitoGFP-positive structures (Figure S2E). Notably, COPII vesicles have been previously shown to localize to MERCS,^43^ and Sar1 loss of function in yeast leads to an increase in the length of these contact sites.^44^ Mitochondrial defects are thus observed upon disruption of COPI and COPII vesicle trafficking in mechanisms independent of Rab6 and Rab1, respectively.

### Mitochondrial defects are also observed in COPA-depleted human cells

**Figure 2:**
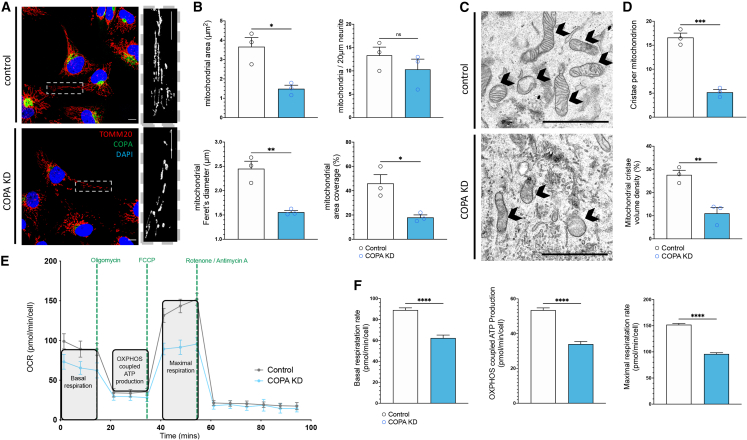
Knockdown of COPA *in vitro* causes fragmented and dysfunctional mitochondria (A) Control and COPA-knockdown (KD) SH-SY5Y cells with labeled with anti-TOMM20 for mitochondrial outer membrane, anti-COPA, and DAPI-stained nuclei. (B) Quantification of median mitochondrial area, median mitochondrial Feret’s diameter, mitochondrial area coverage in the neurites of SH-SY5Y cells, and number of mitochondria per 20 μM neurite show deficits in mitochondrial morphology in COPA-depleted conditions. Each data point represents mean value per coverslip, with ten cell neurites analyzed per coverslip. (C) Transmission electron microscopy (TEM) of control and COPA-KD SH-SY5Y cells show that COPA depletion causes loss of cristae and internal mitochondrial structure. Arrows indicate mitochondria. (D) Quantification shows a reduction of mitochondria with cristae and cristae volume. Individual data points represent mean value per coverslip, with five neurites analyzed per coverslip. (E) Oxygen consumption rate is decreased in COPA-KD cells on Seahorse XF Mito stress test assay. (F) Cell-count-normalized basal respiration rate, OXPHOS-coupled ATP production rate, and maximal respiration rate measured across six wells per condition. Student’s t tests were used to determine significance. Graphs are expressed as mean ± SD and annotated as ^∗^p < 0.05, ^∗∗^p < 0.01, ^∗∗∗^p < 0.001, and ^∗∗∗∗^p < 0.0001. Scale bars, 10 μm (A) and 1 μm (C).

To assess whether our observed mitochondrial phenotypes were conserved between fly and mammalian models, we utilized a doxycycline-inducible, COPI Coat Complex Subunit α (COPA)-targeting, short hairpin RNA (shRNA)-expressing SH-SY5Y cell line.^45^ At 72 h and 96 h after inducing expression of the COPA-targeting shRNA, COPA was depleted from cells, as demonstrated by immunocytochemistry and immunoblotting (Figures S3A and S3B). Mitochondria were labeled by TOMM20 immunostaining, and the mitochondria present within neurite-like projections were quantified at 72 h post doxycycline treatment (Figure 2A). In COPA knockdown (KD) neurites, mitochondrial Feret’s diameter, area, and area coverage were significantly decreased compared to controls (Figure 2B), suggesting that the regulation of the mitochondria network via the COPI is conserved across species.

Mitochondrial dynamics can directly impact on cristae remodeling, which in turn affects mitochondrial bioenergetics.^46^^,^^47^ We performed transmission electron microscopy (TEM) following depletion of COPA and observed mitochondria with much fewer cristae than controls (Figures 2C and 2D). Mitochondrial function was therefore assessed by measuring oxygen consumption rate via the Seahorse Mito stress test assay (Figure 2E). At 72 h post shRNA expression, COPA-KD cells demonstrated a significant decrease in oxygen consumption rate attributable to baseline respiration and ATP-linked and maximal respiratory capacity compared to control cells (Figure 2F), indicative of decreased mitochondria-associated oxidative phosphorylation and ATP production. Loss of COPI therefore causes a significant reduction in oxidative phosphorylation, indicative of severe mitochondrial dysfunction.

### COPI-deficient neurons exhibit abnormalities in axonal ER, Ca^2+^ homeostasis, and ER-associated organelles

**Figure 3:**
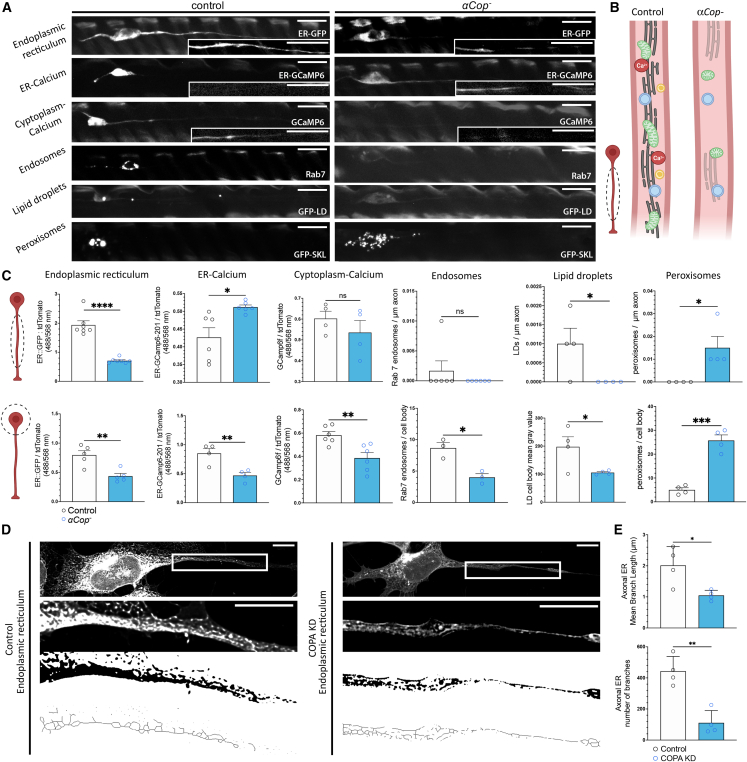
*αCOP* mutant neurons display evidence of ER disruption (A) Genetically encoded organelle markers and Ca^2+^ indicators were expressed in clonal neurons in both control and *αCOP*^−^ mutant backgrounds—*UAS-Sturkopf*::*GFP* (ER), *UAS-ER-GCaMP6-201* (ER Ca^2+^), *UAS-GCaMP6f* (cytosolic Ca^2+^), *UAS-Rab7*::*GFP* (endosomes), *UAS-GFP*::*LD* (lipid droplets), or *UAS-GFP*::*SKL* (peroxisomes)—and imaged at 1 DPE. (B) Depletion of αCOP in axons causes loss of ER, disruption of ER-associated organelles, and loss of cytosolic Ca^2+^ levels in axons. (C) Mean gray values for *UAS-Sturkopf*::*GFP*, *UAS-ER-GCaMP6-201*, *UAS-GCaMP6f*, *UAS-Rab7*::*GFP*, *UAS-GFP*::*LD*, and *UAS-GFP*::*SKL* in either the axon or cell body of control and *αCOP*^−^ mutant in *Drosophila* wing neurons, assessed by manually tracing cell-body regions followed by the “Measure” function in ImageJ. Mean gray values for *UAS-Sturkopf*::*GFP*, *UAS-ER-GCaMP6-201*, and *UAS-GCaMP6f* in the axons of control and *αCOP*^−^ mutant in *Drosophila* wing neurons, assessed by manually tracing axonal regions followed by the “Measure” function in ImageJ. For analysis of *UAS-Rab7*::*GFP*, *UAS-LD*::*GFP* and *UAS-SKL*::*GFP* in axons puncta/μm were measured. Graphs are expressed as mean ± SD. Individual data points represent mean values from individual flies, calculated across 5–10 clones from both wings. (D) ER morphology in the neurites of SH-SY5Y cells using the glycoprotein stain concanavalin A. (E) The number and length of ER branches *in vitro* were reduced following COPA depletion. ER morphology was assessed using the “Skeletonize” function in ImageJ. Student’s t test: ns, not significant; ^∗^p < 0.05, ^∗∗^p < 0.01, ^∗∗∗^p < 0.001, ^∗∗∗∗^p < 0.0001. All scale bars, 10 μm.

Owing to the role of COPI in the retrograde transport of ER-resident proteins from the Golgi network, ER dysfunction has been previously reported as a consequence of COPI deficiency in humans, with a decrease in binding of COPA to ER-resident proteins and an increase in transcriptional and morphological ER stress markers reported.^48^^,^^49^ The *UAS-Sturkopf*::*GFP* reporter^50^ localizes to ER, has been utilized effectively as an ER marker in *Drosophila*, and shows continuous labeling throughout wild-type axons.^12^ We assessed ER levels and distribution with Sturkopf::GFP in both the soma and axons of *αCOP*^−^ mutant neurons, which was significantly decreased in both compartments, indicating severe ER dysfunction (Figures 3A–3C). However, lack of Sturkopf::GFP-positive structure in the axon does not necessarily reflect a lack of ER, as it could be caused by defective localization of the reporter protein to the ER upon COPI depletion. We therefore further assessed ER integrity in the neurites of SH-SY5Y cells using the glycoprotein stain concanavalin A^51^ (Figure 3D). ER morphology was less complex following COPA KD, with fewer branches of tubules that were shorter in length (Figure 3E).

Axonal ER is critical for regulating cellular homeostasis, controlling Ca^2+^ dynamics, and the distribution and function of other organelles, such as endosomes, lipid droplets, and peroxisomes.^30^ We utilized a panel of genetically encoded fluorescent GCaMP indicators to visualize Ca^2+^ compartmentalization in *αCOP*^−^ mutant neurons. Significant decreases in ER-retained Ca^2+^ (ER-GCaMP6-210) and cytosolic Ca^2+^ (GCaMP6f) were observed in the cell body of *αCOP*^−^ mutant neurons compared to controls (Figures 3A–3C). However, in axons a significant increase in ER-retained Ca^2+^ was observed, while cytosolic Ca^2+^ was again decreased (Figures 3A–3C). Taken together, these results indicate that *αCOP*^−^ causes general defects in Ca^2+^ homeostasis and causes inverse effects on somatic and axonal ER Ca^2+^ retention. Reduction in ER tubule number, continuity, and surface area could affect the capacity of ER to take up and release Ca^2+^ in axons, as observed upon loss/overexpression of ER-shaping Reticulon proteins.^52^^,^^53^ This could explain the disparity between somatic and axonal ER Ca^2+^ in *αCOP*^−^ neurons, since there appears to be a greater loss of ER structure in the axon compared to the soma (Figures 2A and 2C), which could lead to axon-specific defects in Ca^2+^ homeostasis.

Since neuronal ER regulates a number of other organelles such as endosomes, lipid droplets, and peroxisomes,^24^ we assessed their distribution and quantity by expressing *UAS-Rab7*::*GFP*, *UAS-GFP*::*LD*^38^ and *UAS-SKL*::*GFP*, respectively in neuronal clones (Figures 3A–3C). A significant decrease in the fluorescence intensity of Rab7::GFP-positive endosomes and GFP::LD-positive lipid droplets was observed in the cell body of *αCOP*^−^ neurons, whereas SKL::GFP-positive peroxisome intensity was increased (Figures 3A–3C). Similar trends were observed in axons, with an increase in peroxisomes observed in *αCOP*^−^ neurons (Figures 3A–3C), suggesting that they could be proliferating in response to mitochondria deficits in this compartment. Rab7::GFP-positive endosomes were rarely observed in control or *αCOP*^−^ axons (Figures 3A–3C). This indicates that COPI transport to the ER plays an important role in Ca^2+^ and organellar homeostatic mechanisms in neurons.

### Mitochondria in COPI-deficient neurons have fewer contact sites with ER and decreased Ca^2+^ and undergo increased lysosomal acidification

**Figure 4:**
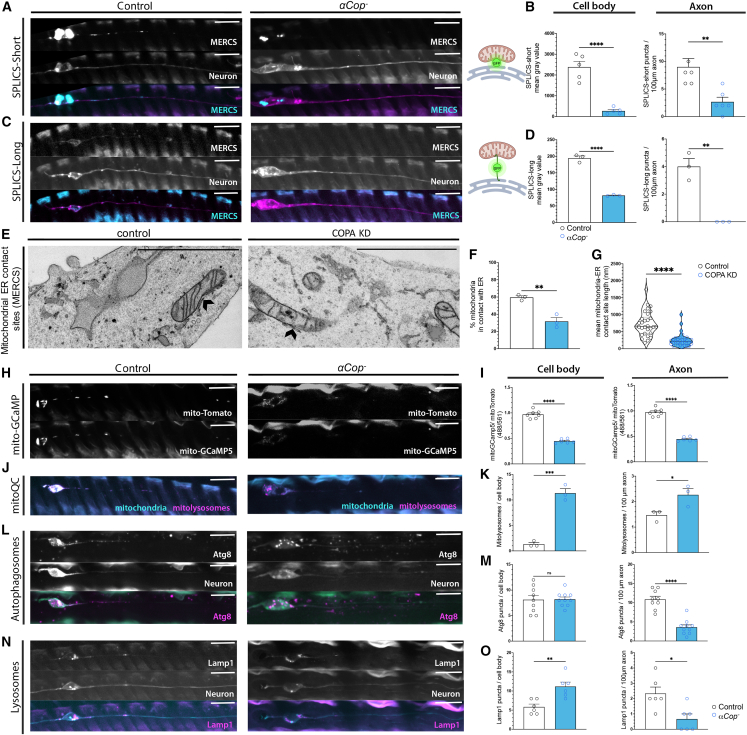
Mitochondria-ER contact sites and mitochondrial Ca^2+^ are decreased while mitophagy is increased in *αCOP* mutant neurons (A) *UAS-SPLICS-short* was expressed in control and *αCOP*^−^ mutant *Drosophila* neuronal clones to visualize mitochondria-ER contact sites (MERCS) of ∼8–10 nm distance. (B) Number of SPLICS-short puncta in control and *αCOP*^−^ mutant cell bodies and axons were significantly reduced compared to control. (C) *UAS-SPLICS-long* was further used to visualize MERCS of ∼40–50 nm distance. (D) SPLICS-long MERCS in *αCOP*^−^ cell bodies and axons were also significantly reduced compared to control. (E) TEM of MERCS in the neurites of COPA KD SY-SY5Y cells and controls. Individual data points represent mean value per coverslip, with five neurites analyzed per coverslip. (F) Percentage of mitochondria in contact with the ER in the neurites was decreased following COPA knockdown (KD). Individual data points represent single contact sites, measured across three coverslips, with five neurites per coverslip. (G) Average length of MERCS in the neurites of COPA-KD SH-SY5Y cells was also decreased compared to control. (H) *UAS-mitoGCaMP5* indicator was specifically expressed in mitochondria to measure luminal Ca^2+^, and *UAS-mito*::*tdTomato* was expressed to indicate the outer membrane. (I) The fluorescence intensity of *UAS-mitoGCaMP5* signal normalized to mito::tdTomato was significantly decreased in αCOP-depleted cell bodies and axons. (J) *UAS-mitoQC*-labeled mitochondria were expressed in control and *αCOP*^−^ mutant neurons, where mCherry-positive, GFP-negative organelles represent acidified mitolysosomes. (K) The number of mitolysosomes observed in 100 μm of axon mitochondria was increased in *αCOP-*depleted neurons. (L) *UAS-mCherry*::*Atg8* was further expressed in neuronal clones to visualize autophagosomes. (M) The number of autophagosomes observed in 100 μm of axon mitochondria was decreased *αCOP*^−^ axons compared to control. (N) *UAS-Lamp1*::*GFP* was used to label neuronal residing lysosomes. (O) The lysosome population observed in 100 μm of axon mitochondria was also depleted in *αCOP*^−^ mutant axons. Each data point represents mean value for an individual fly, calculated across 5–10 clones from both wings. Welch’s t test or Student’s t test was used throughout. Graphs are expressed in mean ± SD and annotated as: ns, not significant; ^∗^p < 0.05, ^∗∗^p < 0.01, ^∗∗∗^p < 0.001, ^∗∗∗∗^p < 0.0001. Scale bars, 10 μm (1 μm in E).

Since the ER is tightly linked to mitochondrial dynamics and function, its dysregulation in neurons upon *αCOP* ablation is likely to be a key upstream determinant in neuronal energetics and survival. We sought to address whether the mitochondrial phenotypes in *αCOP*^−^ mutant neurons are caused by a novel role of COPI at mitochondria or rather as a downstream consequence of general ER disruption. Co-labeling experiments revealed a low co-localization between αCOP or COPA with mitochondria in *Drosophila* wing neurons and SH-SY5Y cells, respectively, with a Pearson’s co-localization coefficient of <0.3 (Figures S4A–S4C). COPI subunits have been shown to localize to MERCS,^54^ which may explain the partial co-localization. To assess whether MERCS are affected by *αCOP*^−^ in *Drosophila* neurons_,_ we employed split-GFP-based contact site sensors (SPLICS) to probe short-range (∼8–10 nm) and long-range (∼45–50 nm) contact sites.^55^^,^^56^ At 1 DPE, a decrease in MERCS normalized to mitochondrial number was observed in the axons of *αCOP*^−^ mutant neurons for reporters of both short- and long-distance contacts (Figures 4A–4D). We also normalized SPLICS to total ER from Sturkopf::GFP-labeled clones and found that although SPLICS levels in the cell body were still significantly lower in *αCOP*^−^ compared to control, in axons ER-normalized SPLICS puncta were unchanged in the mutant (Figure S4D). This indicates that the decrease in axonal MERCS in the *αCOP*^−^ mutant is mainly due to reduced ER structure in this compartment. Since the decrease in SPLICS fluorescence could also be caused by mislocalization of the ER-targeted β-strand 11 of the split GFP molecule caused by COPI deficiency, we also assessed MERCS in the neurites of COPA-KD SH-SY5Y cells by TEM (Figure 4E). Quantification showed a decrease in both percentage of mitochondria in contact with ER (Figure 4F) and the average length of contacts (Figure 4G). Since we also observed a decrease in ER structure in the neurites of these cells (Figures 3D and 3E), this is the most likely cause of the observed decrease in MERCS.

MERCS are important for the exchange of lipids between the two organelles as well as Ca^2+^, which is transferred to mitochondria through IP3R-VDAC (*Drosophila* Itpr/porin) channels from ER to mitochondria.^28^ We used a mitochondrial localized Ca^2+^ indicator (mitoGCaMP5)^32^ to visualize mitochondrial Ca^2+^ in *αCOP*^−^ neurons and compared fluorescence levels of the non- Ca^2+^-dependent mito:Tomato marker (Figure 4H). Normalized fluorescence levels of mitoGCaMP5 were significantly decreased in *αCOP*^−^ cells, indicating that a decrease in MERCS causes a decrease in mitochondrial Ca^2+^ buffering capacity (Figure 4I). We also found that COPA KD decreases mitochondrial Ca^2+^ uptake *in vitro* using the Rhod-2 indicator (Figures S5A and S5B). Loss of COPI therefore impedes MERCS formation in axons and the ability of Ca^2+^ to transfer between the organelles. Intramitochondrial Ca^2+^ is required to activate dehydrogenases coupled to the Krebs cycle and ATP synthase^57^ and may therefore underpin the energetic deficiency we observed in COPA-deficient SH-SY5Y cells.

Significantly compromised mitochondria are prevented from fusing and then degraded through autophagic/lysosomal pathways.^58^ Mito-QC, a GFP-mCherry tandem fusion protein attached to the mitochondrial localization domain of Fis1,^59^^,^^60^ was employed to assess the number of mitochondria undergoing lysosomal degradation. A significant increase in mitolysosomes (mCherry-positive, GFP-negative) was observed in *αCOP*^−^ mutant neurons in both the cell body and axon (Figures 4J and 4K), which was not the consequence of globally increased autophagy, since mCherry::Atg8-positive autophagosome number was not enhanced (Figures 4L and 4M). Dysfunctional mitochondria caused by *αCOP* deletion are therefore sufficient to stimulate the mitophagy pathway in axons. Since mitophagy in neurons has been reported to occur independently of canonical autophagy machinery,^33^^,^^61^^,^^62^ we also assessed the number of lysosomes in the cell. The number of Lamp1-positive lysosomes was also decreased in the axons of *αCOP*^−^ neurons (Figures 4N and 4O), indicating that the increase in mitolysosomes observed in axons is specific to mitochondrial clearance and not a general increase in lysosome number. An increase in mitolysosomes upon KD of the MERCS tether Pdzd8 has previously been reported in neurons^56^; therefore, heightened mitophagy in COPI-depleted conditions is likely downstream of the decrease in MERCS.

**Figure 5:**
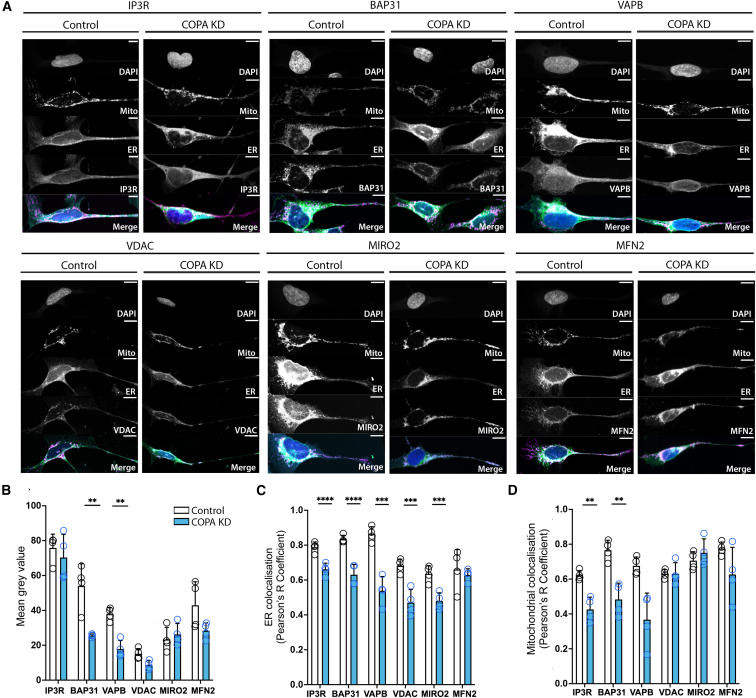
COPA KD *in vitro* causes a decrease in MERC-associated proteins co-localized with the ER and the mitochondria (A) Control and COPA-KD SH-SY5Y cells were co-labeled with DAPI to indicate nuclei, anti-TOMM20 for mitochondrial outer membrane, and concanavalin A::Alexa Fluor 488 for ER, and either anti-IP3R, anti-BAP31, anti-VAPB, anti-VDAC, anti-MIRO2, or anti-MFN2 to label MERCS proteins. (B) Quantification of fluorescence intensity for MERC-associated proteins indicates that depletion of COPA reduces levels of BAP31 and VAPB in neurons. (C) Quantification of MERC-associated proteins in control and COPA-KD SH-SY5Y cells show that COPA depletion causes a mislocalization of IP3R, BAP31, VAPB, VDAC, and MIRO2 with the ER. (D) Quantification of MERC-associated proteins in control and COPA-KD conditions indicates that COPA depletion causes a mislocalization of IP3R, BAP31, and VAPB with mitochondria. Each data point represents mean value per well, with five cells analyzed per well. Two-way ANOVA with FDR correction was used to determine significance. Graphs are expressed as mean ± SD and annotated as ^∗^p < 0.05, ^∗∗^p < 0.01, ^∗∗∗^p < 0.001, and ^∗∗∗∗^p < 0.0001. Scale bars, 10 μm.

MERCS are formed by specific proteins residing on the ER or mitochondria membranes, which are required for tethering and functional channel formation.^23^ We therefore explored whether specific MERCS-associated proteins remained co-localized with the organelles following COPA KD in SH-SY5Y cells by immunocytochemistry (Figure 5A). Interestingly, loss of COPA was found to cause an overall reduction in levels of the ER-resident proteins BAP31 and VAPB, but not in IP3R, VDAC, MIRO2, or MFN2 (Figure 5B). COPA KD also resulted in mislocalization of several MERCS-associated proteins, including BAP31 and VAPB, with both the ER and mitochondrial networks (Figures 5C and 5D). Loss of VDAC and IP3R associated with ER membrane was also observed (Figures 5C and 5D), which may explain mitochondrial Ca^2+^ changes found in both *αCOP*^−^ *Drosophila* neurons and COPA-KD SH-SY5Y cells. These data indicate that the observed reduction of MERCS seen using the SPLICS reporter *in vivo* and at the ultrastructural level *in vitro* is likely caused by depletion and/or mislocalization of key MERCS-associated proteins from both ER and mitochondrial membranes.

### Increasing the number of MERCS in *αCOP*-ablated neurons rescues mitochondrial morphology and Ca^2+^ deficits, enhances the level of ER, and delays neurodegeneration

**Figure 6:**
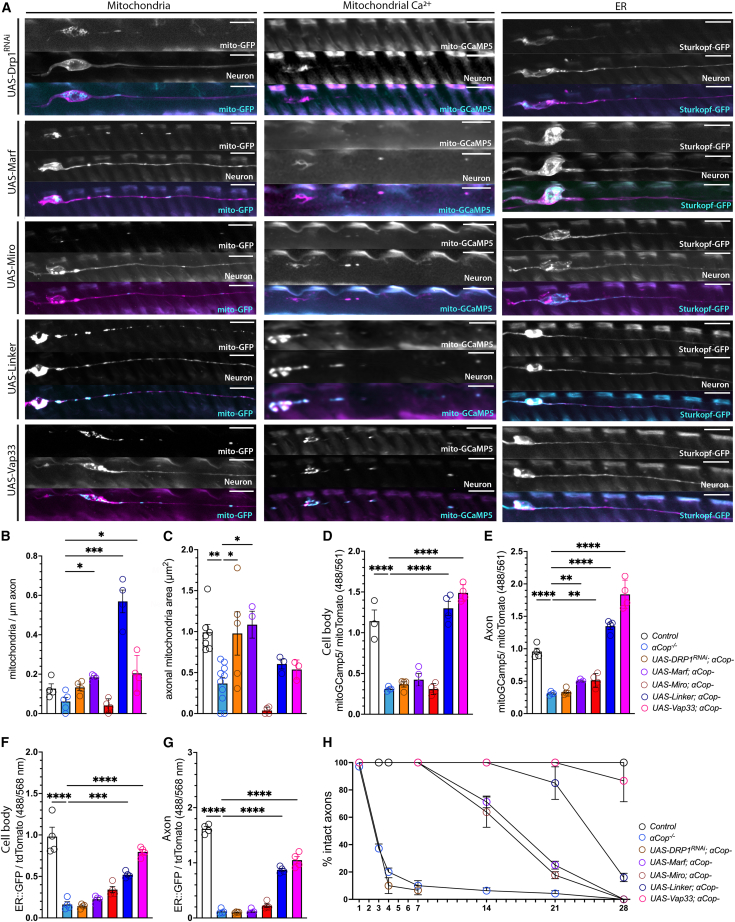
Increasing MERCS is sufficient to rescue ER deficits, mitochondrial Ca^2+^ levels, and neurodegeneration in *αCOP* mutant neurons (A) ER and mitochondrial phenotypes in *αCOP*^−^ mutant *Drosophila* wing neurons at 1 DPE upon Drp1 KD using *UAS-Drp1*^*RNAi*^, Marf overexpression using *UAS-Marf*, Miro overexpression using *UAS-Miro*, artificially tethering the ER and mitochondria using *UAS-Linker*, and increasing MERCS through *UAS-Vap33* overexpression. Markers used: *UAS-Mito*::*GFP* (mitochondria), *UAS-myr*::*tdTomato* (neuronal membrane), *UAS-Sturkopf*::*GFP* (ER), and *UAS-mito*::*GcaMP5* (mitochondrial Ca^2+^ levels). (B) Quantification shows that forcing MERCS in *αCOP*^−^ mutant *Drosophila* wing neurons significantly increases the number of mitochondria in the axon. (C) Changes in mitochondrial dynamics through decreased fission or enhanced fusion significantly rescues the median mitochondrial area in *αCOP*^−^ mutant neuronal clones. (D) Forcing MERCS in *αCOP*^−^ mutant neuronal clones restores mitochondrial Ca^2+^ levels in the cell body. (E) Increasing MERCS in *αCOP*^−^ mutant *Drosophila* wing neurons also restores mitochondrial Ca^2+^ levels in the axon. (F) Forcing MERCS in αCOP-depleted neurons causes increased ER retention in the cell body. (G) Increasing MERCS in *αCOP*^−^ mutant neurons causes increased ER retention in the axon. (H) Enhanced MERCS formation is associated with neuroprotection in αCOP-depleted neurons. Each data point represents mean value for an individual fly, calculated across 5–10 clones from both wings. Data were analyzed by one-way or two-way ANOVA with FDR correction. Graphs are expressed as mean ± SD and annotated as ^∗^p < 0.05, ^∗∗^p < 0.01, and ^∗∗∗∗^p < 0.0001. Scale bars, 10 μm.

To test whether the loss of MERCS was sufficient to cause mitochondrial and ER phenotypes observed in *αCOP*^−^ neurons and COPA-KD cells, we performed epistasis experiments with known modifiers of MERCS formation and mitochondrial dynamics. We first knocked down the mitochondrial fission protein dynamin-related protein 1 (Drp1) and observed a restoration mitochondrial area in *αCOP*^−^ *Drosophila* axons but not in the number of mitochondria present (Figures 6A–6C). The *Drosophila* orthologs of *MFN2* and *MIRO1*/2 (*Marf* and *Miro*) have also been shown to regulate both mitochondrial dynamics and MERCS.^24^^,^^25^^,^^26^^,^^60^ Overexpression of *Marf* restored mitochondrial morphology and number in *αCOP*^−^ axons, whereas overexpression of *Miro* did not (Figures 6A–6C). Next, we overexpressed an artificial ER-mitochondria tether (Linker), which increases MERCS via a mitochondrial localizing domain of mAKAP1 and the ER targeting sequence of yUBC6, fused to monomeric red fluorescent protein.^63^^,^^64^ Forcing MERCS with Linker allowed for a complete rescue of mitochondrial phenotypes in *αCOP*^−^ neurons (Figures 6A–6C). Artificially tethering ER and mitochondria greatly enhanced mitochondrial number above wild-type levels (Figures 6A and 6B), likely due to aberrant mitochondria fission, which has been observed using Linker previously.^64^ Finally, we overexpressed the ortholog of ER-resident protein VAPB (Vap33), which forms MERCS with PTPIP51 in mammals^27^ and mitoguardin in flies.^65^ Vap33 overexpression was also sufficient to rescue mitochondrial number and size in *αCOP*^−^ neurons (Figures 6A–6C).

*αCOP*^−^ caused a significant reduction in mitochondrial Ca^2+^ compared to controls (Figures 4H and 4I). We therefore next assessed the effect of these genetic epistatic manipulations on the function of MERCS to control the transfer of Ca^2+^ from the ER to the mitochondria. Decreasing fission via Drp1 KD was not sufficient to rescue mitochondrial Ca^2+^, whereas Marf overexpression, which also rescued mitochondrial morphology, partially rescued mitochondrial Ca^2+^ levels in the axon (Figures 6A, 6D, and 6E). This indicates that the morphology of mitochondria has little impact on functional MERCS in *αCOP*^−^ axons, since both these genetic backgrounds rescued mitochondrial morphology but only Marf overexpression rescued mitochondrial Ca^2+^. Indeed, overexpression of Marf increased the number of SPLICS-short puncta observed in axons of *αCOP*^−^ neurons, whereas Drp1 KD did not (Figures S6A and S6B). Interestingly, while Miro overexpression was insufficient to rescue mitochondrial morphology, number, or MERCS, it did cause a moderate increase in mitochondrial Ca^2+^ (Figures 6A, 6D, and 6E), despite also failing to significantly increase SPLICS levels in soma or axons (Figures S6A and S6B). Linker or Vap33 overexpression was sufficient to restore both SPLICS levels (Figures S6A and S6B) and mitochondrial Ca^2+^ levels in somatic and axonal compartments of *αCOP*^−^ neurons (Figures 6A, 6D, and 6E), indicating that rescuing MERC formation has a functional effect on mitochondrial Ca^2+^ uptake. We further assessed whether increasing MERCS could also rescue the ER depletion we observed in COPA-deficient models. Strikingly, Sturkopf::GFP fluorescence levels were increased in the soma and axon upon Linker or Vap33 overexpression (Figures 6A, 6F, and 6G), suggesting that adequate mitochondrial contact is needed for ER retention in axons and that a decrease in MERCS contributes to the ER phenotype we observed in *αCOP*^−^ neurons.

To assess what effect these genetic manipulations have on the fate of *αCOP*^−^ neurons, we quantified axon integrity from 1 to 28 DPE (Figure 6H). Drp1 KD in *αCOP*^−^ neurons failed to rescue neurodegeneration (Figure 6H), indicating that restoring mitochondrial morphology alone was not sufficient to rescue neurodegeneration. Overexpression of Marf or Miro was sufficient to significantly delay neurodegeneration, with 100% of *αCOP*^−^ cells intact at 7 DPE and ∼70% remaining at 14 DPE (Figure 6H). Since *Miro* overexpression partially rescued neurodegenerative but not mitochondrial morphology, we interpreted this to mean that the rescue of neurodegeneration is not dependent on mitochondrial dynamics. Linker overexpression blocked neurodegeneration, with 100% of intact axons remaining at 14 DPE and ∼90% at 21 DPE, yet only 20% at 28 DPE (Figure 6H). Although Vap33 overexpression failed to restore mitochondrial area in *αCOP*^−^ axons, it remarkably rescued age-dependent neurodegeneration phenotypes at all age points analyzed (Figure 6H). In control neurons, altering mitochondrial dynamics or increasing MERCS had no effect on neuronal survival with age (Figure S6C). MERCS can therefore maintain axonal homeostasis even in the presence of altered mitochondrial dynamics in COPI-deficient neurons. COPI regulation of MERCS is thus key to maintaining axonal ER, mitochondrial Ca^2+^ uptake, and, ultimately, neuronal survival.

## Discussion

Axonal ER is highly important for neuronal function, and proteins that regulate tubular ER organization can cause a range of neuropathies.^12^^,^^66^ Contacts between ER and mitochondrial membranes facilitate lipid and Ca^2+^ homeostasis as well as mitochondrial dynamics, and neurons are particularly sensitive to perturbations in these processes. Here, we have demonstrated that the COPI is a crucial link between the ER, mitochondria, and axonal homeostasis.

In *S*. *cerevisiae*, there have been conflicting reports on the effect of COPI inhibition on mitochondrial morphology. A temperature-sensitive *βCOP* mutant allele caused aggregated mitochondria at restrictive temperatures,^14^ whereas another study found no effect on mitochondrial morphology in *γCOP* mutants at permissive or restrictive temperatures.^13^ The same study also found no effect on mitochondrial morphology in *C*. *elegans* muscle tissue upon *βCOP* KD. We have demonstrated that COPI inhibition causes significant alterations in mitochondrial form and function in *Drosophila* neurons and human neuroblastoma cells.

Microdomains of PI(4)P on *trans*-Golgi network (TGN)-derived vesicles are recruited to MERCS to promote mitochondrial fission in a mechanism regulated by ARF1 and its effector, phosphatidylinositol 4-kinase IIIβ (PI(4)KIIIβ).^15^ ARF1 and PI(4)KIIIβ localize to these TGN vesicles at mitochondrial constriction sites, which are in contact with both ER and lysosomes and are required for DRP1-mediated mitochondrial fission. In the fungus *C*. *albicans*, *Arf1* deletion leads to a reactive oxygen-species-dependent increase in MERCS.^67^ However, the role of COPI in MERCS regulation had not been previously explored. Here, we demonstrated that COPI deficiency results in a decrease in axonal MERCS and that overexpression of MERCS proteins is sufficient to rescue neurodegeneration independent of mitochondrial morphology. These findings point toward the importance of MERCS in maintaining axonal homeostasis and the important role of COPI in maintaining them.

COPI inhibition was previously shown to cause respiratory growth in yeast via COPI-dependent delivery of nuclear mRNAs encoding mitochondrial proteins (mMPs) to mitochondria for local translation,^14^ including mRNA-encoding FIS1, which forms MERCS with BAP31.^68^ COPI binding of specific mRNAs has also been observed in neurons, and COPA-bound mRNAs are enriched for axonal transcriptome members.^69^ Although neither FIS1 nor other contact site mRNAs were identified in the latter study, it is possible that the decrease in MERCS we observed could be caused by a decrease in delivery of mMPs to mitochondria or MERCS for local translation. This phenomenon, mediated by a range of RNA-binding proteins, is a well-known feature of neuronal homeostasis^70^ and includes the delivery of mRNA-encoding proteins that regulate MERCS^71^ and mitophagy.^72^

We found that alterations in mitochondrial morphology are not responsible for the degeneration of COPI-deficient neurons, since Drp1 knockdown was sufficient to rescue mitochondrial morphology, but not neurodegeneration, in our *Drosophila* wing model. The partial rescue of degeneration in *αCOP* mutant neurons by overexpression of Marf is therefore likely due to restoring MERCS. Previous studies found that in MFN2-knockout cells, reintroducing mitochondria-targeted MFN2 was sufficient to rescue mitochondrial morphology, whereas only ER-localized MFN2 was sufficient to restore aberrant MERCS, ER morphology, mitochondrial Ca^2+^ uptake, and bioenergetics,^25^^,^^73^ which are phenotypes similar to those we observed in COPI-deficient cells. The full rescue of neurodegeneration caused by Vap33 overexpression as well as the synthetic Linker in *αCOP* mutant neurons indicates that availability of ER-resident MERCS proteins at the ER is a major factor in the degeneration of COPI-deficient cells. VAPB also mediates contact sites between the ER and peroxisomes,^74^ an organelle that was also mislocalized in the *αCOP* mutant. Although the rescue by the mitochondria-ER Linker indicates that contact between these two organelles is the driving force behind the mutant phenotypes we observed, Vap33 overexpression provided enhanced rescue of axonal integrity at 28 DPE compared to Linker, and this could be explained by enhanced ER contact sites with other organelles.

Whether and how COPI vesicles deliver proteins such as VAPB directly to MERCS to facilitate their formation in a targeted manner warrants further investigation, since VAPB does not possess a COPI recognition motif. In fact, of the multiple ER-resident proteins we found to be mislocalized from the ER in COPA-KD cells, only BAP31 possesses the C-terminal dilysine motif recognized by COPI vesicles. Recent evidence indicates that VAPB tethers at MERCS are formed by a highly dynamic pool of ER-localized VAPB, facilitating rapid remodeling.^75^ The amyotrophic lateral sclerosis-associated VAPB P56S mutation, residing in the MSP domain responsible for binding to PTPIP51, causes VAPB to aggregate and results in a decrease in the mobility of VAPB at MERCS. Interestingly, the same mutation results in a decrease in co-immunoprecipitation of a number of COPI subunits with VAPB,^76^ indicating crosstalk between COPI and VAPB-mediated MERCS. VAPB was first identified as a protein involved in intra-Golgi trafficking, and its inhibition leads to accumulation of COPI vesicles *in vitro*.^77^ Mitochondrial depletion in axons has also been previously observed in Vap33-deficient neurons,^78^ again demonstrating an interaction between COPI, ER, and mitochondria.

Overexpression of Linker, Vap33, and, to a lesser extent, Marf, revealed the intrinsic link between mitochondria and ER in maintaining axonal integrity. These genetic manipulations rescued not only MERCS and mitochondrial Ca^2+^ levels but also the levels of Sturkopf::GFP-labeled ER in the axon. Mitochondria and ER tubules both rely on kinesin-1 for anterograde motility,^79^^,^^80^ and the two organelles have been observed moving in tandem along acetylated microtubules.^81^ In axons, impeding ER interaction with microtubules by disruption of the ER-shaping protein Reticulon 2 leads to a decrease in axonal transport of dense core and synaptic vesicles.^82^ Interaction between mitochondria and ER could thus be important for the efficient tethering of both organelles to microtubules and their motility along the axon. It would be interesting to see whether enhancing COPI function would be sufficient to increase MERC formation or axonal transport and the dependence of this on motor and adapter proteins.

COPI function has previously been linked to the neurological disorders spinal muscular atrophy (SMA) and Alzheimer’s disease (AD). Several rare, highly penetrant SNPs within COPI subunit genes have been associated with AD.^83^ These variants have been demonstrated to affect amyloid precursor protein (APP) processing to produce Aβ40 and Aβ42.^84^ Furthermore, a genome-wide association has been made between COPG2 and the Aβ42/40 ratio in AD patient blood plasma, a strong biomarker for amyloid pathogenesis.^85^ MERCS are also affected in AD, with a decrease in VAPB-PTPIP51 interactions observed in temporal cortex pyramidal neurons.^86^ APP processing occurs at MERCS, and disruption of these membranes can affect the production of Aβ peptides.^87^ Since we have demonstrated that COPI dysfunction results in a decrease in MERCS, this could be the cause of aberrant APP processing and AD risk in carriers of COPI subunit SNPs. Indeed, modulation of MERCS via the artificial linker used in this study improves the fitness of Aβ42-expressing *Drosophila* models of AD.^64^ However, knocking down *Pdzd8*, which regulates MERCS and may reside on both the outer mitochondrial membrane and ER, also improves the fitness of Aβ42-expressing flies.^88^ The reason for this discrepancy is unclear but could be due to tissue specificity, since artificial linker overexpression was performed ubiquitously,^64^ whereas *Pdzd8* KD was performed pan-neuronally.^88^ COPA has been shown to physically interact with the SMA-causing survival motor neuron (SMN) protein and transport it to the growth cone to support neurite outgrowth.^45^^,^^89^ SMN loss of function also disrupts COPI-dependent trafficking.^90^ Mitochondrial respiratory capacity and size is affected in multiple mouse models of SMA.^91^^,^^92^ SMN deficiency can also cause aberrant splicing of the gene *Stasimon*,^93^ which encodes an ER-resident protein that is transported by the COPI and forms MERCS with the mitochondrial porin ion channel VDAC.^94^ Together, this evidence supports our hypothesis for disruption of COPI-dependent transport of ER-resident MERCS proteins leading to severe neuronal dysfunction.

Our study demonstrates the importance of COPI in the maintenance of neuronal ER, mitochondria, and overall axonal survival, providing evidence for the importance of Golgi-ER transport in axons.^95^ Since promoting MERCS was sufficient to rescue COPI-deficiency-induced neurodegeneration, it also re-emphasizes the importance of these functional contact sites in maintaining neuronal health.

### Limitations of the study

While we have demonstrated that promoting MERCS is sufficient to maintain axonal ER in the absence of COPI trafficking, this study has not addressed the entire mechanism by which this occurs. As alluded to above, we believe that microtubule association of mitochondria and ER plays a role, but this remains to be proven. Our work in this study was limited to the role of COPI in *Drosophila* neurons and human neuron-like cells. Although beyond the scope of this study, it would be interesting to investigate whether our observed findings in this study of COPI in neurons are extendable to other cell types and organisms. We also observed that deletion of Sar1 caused a striking absence of mitoGFP labeling in neuronal clones; however, any loss of mitochondria should be investigated at much greater resolution. Investigation into COPII-mediated control of axonal mitochondria warrants further investigation.

## STAR★Methods

### Key resources table

**Table:** 

REAGENT or RESOURCE	SOURCE	IDENTIFIER
**Antibodies**		

Anti-TOMM20	Proteintech	11802-1-AP
Anti-COPA	Santa Cruz	sc-398099
Anti-Bap31	Santa Cruz	sc-393810
Anti-IP3R1	Abcam	ab264281
Anti-MIRO2	Abcam	ab224089
Anti-MFN2	Abcam	ab56889
Anti-VAPB	Abcam	ab196487
Anti-VDAC	Abcam	ab14734
Goat anti-Mouse IgG (H + L) Cross-Adsorbed Secondary Antibody, Alexa Fluor™ 488	ThermoFisher Scientific	Invitrogen A11001
IgG (H + L) Cross-Adsorbed Rabbit anti-Goat, Alexa Fluor™ 568	ThermoFisher Scientific	Invitrogen A11079

**Chemicals, peptides, and recombinant proteins**		

Halocarbon Oil 27	Sigma	Cat#H8773
Ethyl methanesulfonate (EMS)	Sigma	Cat#M0880
Vectashield	Vector Laboratories	Cat#H1000
Rhod-2	Abcam	ab142780
Pluronic	ThermoFisher Scientific	Gibco 24040032
Mitotracker Deep Red	ThermoFisher Scientific	Invitrogen M22426
Concanavalin A - Alexa Fluor 488	ThermoFisher Scientific	Invitrogen C11252
Hoechst 33342, Trihydrochloride, Trihydrate – 10 mg/mL Solution in Water	ThermoFisher Scientific	Cat#H3570
DMEM/F-12, GlutaMAX™	ThermoFisher Scientific	Gibco 31331093

**Critical commercial assays**		

Seahorse XF Cell Mito Stress Test Kit	Agilent Technologies	Cat#103015-100
Seahorse XF96 Cell Culture Microplate	Agilent Technologies	Cat#101085-004
Seahorse Xfe96 Extracellular Flux Assay Kits	Agilent Technologies	Cat#102601-100

**Experimental models: Cell lines**		

COPA shRNA SH-SY5Y	Androphy Lab^45^	N/A

**Experimental models: Organisms/strains**		

*OK371-Gal4*	BDSC	RRID:BDSC_26160
*10xUAS-IVS-myr*::*tdTomato*	BDSC	RRID:BDSC_32222
*5xUAS-mito*::*GFP*	BDSC	RRID:BDSC_8442
*5xUAS-GFP*::*SKL*	BDSC	RRID:BDSC_28880
*FRT2A*	BDSC	RRID:BDSC_1997
*FRT82B*	BDSC	RRID:BDSC_2035
*tub-Gal80*	BDSC	RRID:BDSC_5135
ey-FLP	BDSC	RRID:BDSC_5577
*asense-FLP2e*	Freeman Lab^34^	N/A
*UAS-αCOP*.*ORF*.*3xHA*	FlyORF^36^	Stock #F001833
*Pbac{fTRG01193*.*sfGFP-TVPTBF}VK00002*	VDRC^96^	VDRC:v318793
*FRT82B*, *γCop^10^*	BDSC	RRID:BDSC 29706
*Rab6*^*EP2397*^, *FRT40A*	Kyoto DGGR	Stock # 114449
*FRT82B*, *Rab1*^[*S147213*]^	BDSC	RRID:BDSC 37735
*FRT82B*, *Sar1*^[*11-3−*^*^63^*^]^	BDSC	RRID:BDSC 53710
*UAS-Rab6*:*YFP*	BDSC	RRID:BDSC 23251
*20xUAS-mito*::*GcaMP5*	Freeman Lab^32^	N/A
*UAS-Gcamp6f*	BDSC	RRID:BDSC 42747
*UAS-ER-Gcamp6-210*	BDSC	RRID:BDSC 91396
*UAS-GFP*::*LD*	Welte Lab^97^	N/A
*UAS-Sturkopf*::*GFP*	O’Kane Lab^12^	N/A
*UAS-GFP*::*SKL*	BDSC	N/A
*UAS-Rab7*::*YFP*	BDSC	RRID:BDSC 42705
*UAS-mitoQC*	Whitworth lab^60^	N/A
*UAS-SPLICS-short*	Whitworth lab^56^	N/A
*UAS-SPLICS-long*	Vagnoni lab	N/A
*UAS-mCherry*::*Atg8*	Neufeld Lab^98^	N/A
*UAS-Linker*	Martins Lab^64^	N/A
*UAS-FLAG*::*Vap33*::*HA*	BDSC	RRID:BDSC 39682
*UAS-Marf*	BDSC	RRID:BDSC 67157
*UAS-Miro*	BDSC	RRID:BDSC 51646
*UAS-Drp1*^*RNAi*^	BDSC	RRID:BDSC 51483
autosome deficiencies	BDSC	https://bdsc.indiana.edu/stocks/df/dfkit-info.htm

**Software and algorithms**		

Zen Blue	Zeiss	www.zeiss.com
Leica Application Suite (LAS)-X Core	Leica	www2.leicabiosystems.com
Harmony	Revvity	https://www.perkinelmer.com/uk/product/harmony-4-8-office-hh17000001
Prism9	Graph Pad	www.graphpad.com
Seahorse Wave	Agilent Technologies	https://www.agilent.com/zh-cn/product/cell-analysis/real-time-cell-metabolic-analysis/xf-software/seahorse-wave-desktop-software-740897
ImageJ	Version 1.53s	www.ImageJ.nih.gov

### Resource availability

#### Lead contact

Further information and requests for resources and reagents should be directed to and will be fulfilled by the Lead Contact, Gaynor Smith (smithga@cardiff.ac.uk).

#### Materials availability

All materials generated in this study will be shared upon request.

### Experimental model and subject participant details

#### Drosophila maintenance & EMS mutagenesis and generation of MARCM clones

All experimental stocks were reared at 25°C on cornmeal, molasses and yeast medium. As no phenotypic differences were observed between sexes, flies of both sexes were used in equal numbers and data pooled throughout. EMS mutagenesis was performed as previously published.^32^^,^^33^ Briefly, male flies were starved on H_2_O-soaked filter paper for 8 h then transferred to filter paper soaked in 25mM EMS, 1% sucrose for 12 h. Flies were recovered on non-EMS medium for 12 h before mating. F1 progeny from mutagenized males were aged for 7 days before screening. Fluorescently labeled, homozygous mutant glutamatergic neuronal clones were generated using the MARCM system and a flippase source under control of the *asense* gene promoter, as previously described.^32^^,^^34^ Briefly, fluorescent reporters under UAS-GAL4 control are supressed by a ubiquitously expressed tubulin-GAL80, which resides distal to an FRT site on chromosome arm 3L. Upon flippase-mediated, site-specific recombination during mitosis, 3L chromatid arms are recombined at reciprocal FRT sites, resulting in tubulin-GAL80 carrying chromatids being exchanged for EMS-induced mutation-carrying chromatids. Upon cell division, a proportion of daughter neuronal precursor cells are thus homozygous for the EMS-induced mutation, but lacking tubulin-GAL80 and therefore repression of fluorescent reporter expression In this way, a small subset of glutamatergic neurons become homozygous for the EMS-induced mutation and fluorescently labeled for a reporter of choice, in an otherwise heterozygous mutant, unlabelled animal.

#### Cell culture

Human neuroblastoma SH-SY5Y cells containing a stable integration of a doxycycline (dox)-inducible, COPA targeting shRNA^45^ were gifted by Androphy lab, Indiana University, Indianapolis, USA. Cells were cultured in DMEM:F12 (Gibco 11320033) and were selected in media containing final concentrations of 4 μg/mL blasticidin and 0.75 μg/mL puromycin. Cells were routinely cultured in 10% FBS supplemented media but serum was reduced to 1% FBS for 2 passages prior to plating for experiments, to encourage proliferation of neuronal-like cells over endothelial-like cells. For experiments, cells were seeded onto laminin-coated coverslips or glass-bottomed, 96-well Sensoplates (Greiner) at 2.5x10^4^ cells/cm^2^ in DMEM:F12, 1% FBS. After 24 h, cells were treated with a final concentration of 4 μg/mL dox in DMEM:F12, 0.5% FBS for 72 h prior to experimentation. Media was changed to fresh 4 μg/mL dox in DMEM:F12, 0.5% FBS every 24 h.

### Method details

#### Immunocytochemistry

72 h after dox treatment, cells were fixed in 4% paraformaldehyde in DMEM:F12, 0.5% FBS for 20 min at 37°C. Cells were washed 3 times in PBS, permeabilised and blocked for 60 min at RT in 10% normal goat serum, PBS PBS-Triton X 0.1%. All primary antibody incubations (1:100 dilution in 10% normal goat serum, PBS PBS-Triton X 0.1%) were performed overnight at 4°C, followed by 3 washes in PBS. Secondary antibody incubations (1:500 dilution in PBS) were performed for 60 min at room temperature, followed by 3 washes in PBS.

#### Confocal microscopy

All *Drosophila* wing neuron and leg NMJ microscopy was performed on a Zeiss Cell Observer confocal microscope using 488 nm or 568 nm laser lines, a Yakagawa spinning disc, 63x, 1.4 numerical aperture oil objective and Axiocam 503 monochromatic camera using Zen Blue software (Zeiss). Exposure time and laser intensity varied considerably depending on the reporters imaged and are available upon request. With the exception of mitochondrial motility and Ca^2+^ reporters, wings and legs were dissected with microdissection scissors, mounted in halocarbon oil between a microscope slide and coverslip and imaged immediately.^35^ For imaging of mitochondrial motility and Ca^2+^ reporters, *Drosophila* were live-mounted between a microscope slide and a tape-spaced coverslip to create a custom-sized chamber.^40^ Wings were painted down with halocarbon oil and imaged immediately for 240 s at 1 frame per second. Kymographs were generated using the “*Velocity Measurement*” function on ImageJ. Motile mitochondria were classified as those moving >2μm without changing direction during the 180 s recorded, measured by the “*Manual Tracking*” plugin.

For SH-SY5Y cells, coverslips were mounted onto microscope slides in VECTASHIELD PLUS mounting medium, whereas Sensoplates were imaged directly on a Leica SP8 confocal microscope using Leica Application Suite X software in Lightning deconvolution mode, using a 63x, 1.4 numerical aperture oil objective and HyD hybrid detector. 405 nm, 488 nm, 552 nm and 638 nm laser lines were used in line sequential mode (line average = 3).

#### Organelle analysis

Mitochondrial morphology and number were analyzed as previously described.^35^ Briefly, mitochondria and axonal regions were manually traced in ImageJ and shape descriptors were quantified using the “*Measure*” function. mitoGCamp fluorescence intensity measurements were normalised to Ca^2+^-independent mitoTomato intensity to account for potential differences in mitochondrial protein import. SPLICS puncta were counted manually and normalized to either mean mitochondrial number/100μm axon or mean Sturkopf:GFP fluorescence intensity to control for differences in abundance of the two organelles in axons. In the soma, SPLICS signal was quantified by manual tracing of the cell body, followed by “Measure” to extract mean gray value of the reporter. Mitolysosomes, autophagosomes, lysosomes, endosomes, lipid droplets and peroxisomes were quantified in cell bodies by manual tracing of somatic regions in ImageJ and manual counting of organelles. In axons, puncta were counted manually for 100μm of axon proximal to the cell body. Area coverage measurements were calculated as the sum of organelle areas divided by the area of the axon or synaptic bouton surveyed, multiplied by 100.

In SH-SY5Y neurites, mitochondrial were analyzed by manually thresholding the images in ImageJ and made binary. “*Analyze Particles*” was used to automatically detect the mitochondria and extract shape descriptors. Mitochondrial number per 20 μm of neurite was counted manually. ER morphology was assessed as previously described.^99^ Briefly, images were manually thresholded and made binary in ImageJ. Binary images were skeletonized by the “*Skeletonize*” function, followed by the “*Analyze Skeleton*” plugin to extract branch number and size.

For Rhod-2 Ca^2+^ Imaging of SH-SY5Y cells a modified protocol from^96^ was performed. Cells were seeded in 96-well Sensoplates and dox-induced as above. 72 h after dox treatment, cells were loaded with 5 μM rhod-2 in pluronic and 500nM Mitotracker Deep Red in Ca^2+^- free HBSS with or without 20 μM Ru360 to inhibit the mitochondria calcium uniporter (MCU). Cells were incubated at room temperature for 30 min, followed by 37°C for dye de-esterification. Cells were then treated with 0, 1 or 2 mM CaCl_2_ supplemented Ca^2+^-free Hank’s Balanced Salt Solution and imaged automatically on an Opera Phenix (Revvity) at 37°C, 5% CO_2_ with a 40x water-immersion objective. Mitotracker Deep Red positive structures (640 nm excitation) were segmented and emission after excitation at 561 nm in these segments was taken as the value of rhod-2 fluorescence in mitochondria, using Harmony analysis software (Revvity).

#### Transmission Electron Microscopy

Cells were seeded onto laminin-coated coverslips at 2.5x10^4^ cells/cm^2^ in DMEM:F12,1% FBS and treated with a final concentration of 4 μg/mL dox in DMEM:F12, 0.5% FBS after 24 h 72 h after dox treatment, cells were fixed in 2% glutaraldehyde 0.1 M Sodium Cacodylate buffer, pH 7.4 for 60 min at 4°C. Cells were dehydrated, stained with osmium tetroxide, uranyl acetate and lead citrate, embedded in resin, sectioned and mounted onto copper grids. Sections were stained with uranyl acetate and lead citrate before imaging on an FEI Tecnai 12 120 kV BioTwin Spirit TEM. Mitochondrial cristae density was extracted by manual tracing over mitochondrial outer and inner membranes in ImageJ, followed by the “*Measure*” function. Number of cristae per mitochondria was counted manually.

#### Seahorse XF cell Mito Stress Test

Cells were seeded at 2.5x10^5^ cells in 10 cm^2^ dishes in DMEM:F12, 1% FBS and were treated with a final concentration of 4 μg/mL dox in DMEM:F12, 0.5% FBS after 24 h 48 h later, cells were seeded at 4x10^4^ cells/well of a Seahorse XF Pro M Microplate in DMEM:F12, 0.5% FBS. 24 h later, media was replaced with Seahorse XF DMEM pH 7.4, supplemented with glucose, pyruvate and glutamine. Cells were then incubated in a 37°C, 0% CO_2_ incubator for 60 min prior to the assay. An XFe96 Sensor Cartridge was hydrated with 200 μL XF Calibrant overnight in a 37°C, 0% CO_2_ incubator prior to the assay. Oligomycin (1.5 μM final concentration), FCCP (2.0 μM final concentration) and rotenone/antimycin A (0.5 μM final concentration) were sequentially injected from ports A, B & C during the assay. Data was collected and analyzed on Seahorse Wave Desktop Software (Agilent).

### Quantification and statistical analysis

All statistical analysis was performed in GraphPad Prism 9. Information regarding representation of individual data points, averages and error bars, n number and statistical tests applied are included in each figure legend. Data were subjected to normality testing in GraphPad Prism 9 to determine the use of parametric/non-parametric tests. Standard deviations were assessed prior to testing to determine whether to assume equal SD between groups.

## Data Availability

• Data reported in this work will be made available from the lead contact upon request. • This paper does not report original code. • Any additional information required to reanalyse the data reported in this work is available from the lead contact upon request.

## Supplemental information

Document S1. Figures S1–S6

Document S2. Article plus supplemental information

## References

[1] Waters M.G., Serafini T., Rothman J.E. (1991). “Coatomer”: a cytosolic protein complex containing subunits of non-clathrin-coated Golgi transport vesicles. Nature.

[2] Letourneur F., Gaynor E.C., Hennecke S., Démollière C., Duden R., Emr S.D., Riezman H., Cosson P. (1994). Coatomer is essential for retrieval of dilysine-tagged proteins to the endoplasmic reticulum. Cell.

[3] Aridor M., Bannykh S.I., Rowe T., Balch W.E. (1995). Sequential coupling between COPII and COPI vesicle coats in endoplasmic reticulum to Golgi transport. J. Cell Biol..

[4] Scales S.J., Pepperkok R., Kreis T.E. (1997). Visualization of ER-to-Golgi Transport in Living Cells Reveals a Sequential Mode of Action for COPII and COPI. Cell.

[5] Weigel A.V., Chang C.-L., Shtengel G., Xu C.S., Hoffman D.P., Freeman M., Iyer N., Aaron J., Khuon S., Bogovic J. (2021). ER-to-Golgi protein delivery through an interwoven, tubular network extending from ER. Cell.

[6] Wong-Dilworth L., Rodilla-Ramirez C., Fox E., Restel S.D., Stockhammer A., Adarska P., Bottanelli F. (2023). STED imaging of endogenously tagged ARF GTPases reveals their distinct nanoscale localizations. J. Cell Biol..

[7] Serafini T., Orci L., Amherdt M., Brunner M., Kahn R.A., Rothman J.E. (1991). ADP-Ribosylation factor is a subunit of the coat of Golgi-derived COP-coated vesicles: A novel role for a GTP-binding protein. Cell.

[8] Popoff V., Langer J.D., Reckmann I., Hellwig A., Kahn R.A., Brügger B., Wieland F.T. (2011). Several ADP-ribosylation Factor (Arf) Isoforms Support COPI Vesicle Formation. J. Biol. Chem..

[9] Presley J.F., Ward T.H., Pfeifer A.C., Siggia E.D., Phair R.D., Lippincott-Schwartz J. (2002). Dissection of COPI and Arf1 dynamics in vivo and role in Golgi membrane transport. Nature.

[10] Lindsey J.D., Ellisman M.H. (1985). The neuronal endomembrane system. III. The origins of the axoplasmic reticulum and discrete axonal cisternae at the axon hillock. J. Neurosci..

[11] Terasaki M., Slater N.T., Fein A., Schmidek A., Reese T.S. (1994). Continuous network of endoplasmic reticulum in cerebellar Purkinje neurons. Proc. Natl. Acad. Sci. USA.

[12] Yalçın B., Zhao L., Stofanko M., O’Sullivan N.C., Kang Z.H., Roost A., Thomas M.R., Zaessinger S., Blard O., Patto A.L. (2017). Modeling of axonal endoplasmic reticulum network by spastic paraplegia proteins. Elife.

[13] Ackema K.B., Hench J., Böckler S., Wang S.C., Sauder U., Mergentaler H., Westermann B., Bard F., Frank S., Spang A. (2014). The small GTPase Arf1 modulates mitochondrial morphology and function. EMBO J..

[14] Zabezhinsky D., Slobodin B., Rapaport D., Gerst J.E. (2016). An Essential Role for COPI in mRNA Localization to Mitochondria and Mitochondrial Function. Cell Rep..

[15] Nagashima S., Tábara L.C., Tilokani L., Paupe V., Anand H., Pogson J.H., Zunino R., McBride H.M., Prudent J. (2020). Golgi-derived PI(4)P-containing vesicles drive late steps of mitochondrial division. Science.

[16] Godi A., Pertile P., Meyers R., Marra P., Di Tullio G., Iurisci C., Luini A., Corda D., De Matteis M.A. (1999). ARF mediates recruitment of PtdIns-4-OH kinase-beta and stimulates synthesis of PtdIns(4,5)P2 on the Golgi complex. Nat. Cell Biol..

[17] Bottanelli F., Kilian N., Ernst A.M., Rivera-Molina F., Schroeder L.K., Kromann E.B., Lessard M.D., Erdmann R.S., Schepartz A., Baddeley D. (2017). A novel physiological role for ARF1 in the formation of bidirectional tubules from the Golgi. Mol. Biol. Cell.

[18] Giacomello M., Pyakurel A., Glytsou C., Scorrano L. (2020). The cell biology of mitochondrial membrane dynamics. Nat. Rev. Mol. Cell Biol..

[19] Poole A.C., Thomas R.E., Andrews L.A., McBride H.M., Whitworth A.J., Pallanck L.J. (2008). The PINK1/Parkin pathway regulates mitochondrial morphology. Proc. Natl. Acad. Sci. USA.

[20] Pickles S., Vigié P., Youle R.J. (2018). Mitophagy and Quality Control Mechanisms in Mitochondrial Maintenance. Curr. Biol..

[21] König T., Nolte H., Aaltonen M.J., Tatsuta T., Krols M., Stroh T., Langer T., McBride H.M. (2021). MIROs and DRP1 drive mitochondrial-derived vesicle biogenesis and promote quality control. Nat. Cell Biol..

[22] Devine M.J., Birsa N., Kittler J.T. (2016). Miro sculpts mitochondrial dynamics in neuronal health and disease. Neurobiol. Dis..

[23] Wilson E.L., Metzakopian E. (2021). ER-mitochondria contact sites in neurodegeneration: genetic screening approaches to investigate novel disease mechanisms. Cell Death Differ..

[24] Modi S., López-Doménech G., Halff E.F., Covill-Cooke C., Ivankovic D., Melandri D., Arancibia-Cárcamo I.L., Burden J.J., Lowe A.R., Kittler J.T. (2019). Miro clusters regulate ER-mitochondria contact sites and link cristae organization to the mitochondrial transport machinery. Nat. Commun..

[25] de Brito O.M., Scorrano L. (2008). Mitofusin 2 tethers endoplasmic reticulum to mitochondria. Nature.

[26] Han S., Zhao F., Hsia J., Ma X., Liu Y., Torres S., Fujioka H., Zhu X. (2021). The role of Mfn2 in the structure and function of endoplasmic reticulum-mitochondrial tethering *in vivo*. J. Cell Sci..

[27] De Vos K.J., Mórotz G.M., Stoica R., Tudor E.L., Lau K.-F., Ackerley S., Warley A., Shaw C.E., Miller C.C.J. (2012). VAPB interacts with the mitochondrial protein PTPIP51 to regulate calcium homeostasis. Hum. Mol. Genet..

[28] Szabadkai G., Bianchi K., Várnai P., De Stefani D., Wieckowski M.R., Cavagna D., Nagy A.I., Balla T., Rizzuto R. (2006). Chaperone-mediated coupling of endoplasmic reticulum and mitochondrial Ca2+ channels. J. Cell Biol..

[29] Namba T. (2019). BAP31 regulates mitochondrial function via interaction with Tom40 within ER-mitochondria contact sites. Sci. Adv..

[30] Öztürk Z., O’Kane C.J., Pérez-Moreno J.J. (2020). Axonal Endoplasmic Reticulum Dynamics and Its Roles in Neurodegeneration. Front. Neurosci..

[31] Wang J., Fourriere L., Gleeson P.A. (2020). Local Secretory Trafficking Pathways in Neurons and the Role of Dendritic Golgi Outposts in Different Cell Models. Front. Mol. Neurosci..

[32] Smith G.A., Lin T.-H., Sheehan A.E., Van der Goes van Naters W., Neukomm L.J., Graves H.K., Bis-Brewer D.M., Züchner S., Freeman M.R. (2019). Glutathione S-Transferase Regulates Mitochondrial Populations in Axons through Increased Glutathione Oxidation. Neuron.

[33] Lin T.-H., Bis-Brewer D.M., Sheehan A.E., Townsend L.N., Maddison D.C., Züchner S., Smith G.A., Freeman M.R. (2021). TSG101 negatively regulates mitochondrial biogenesis in axons. Proc. Natl. Acad. Sci. USA.

[34] Neukomm L.J., Burdett T.C., Gonzalez M.A., Züchner S., Freeman M.R. (2014). Rapid in vivo forward genetic approach for identifying axon death genes in <em>Drosophila</em&gt. Proc. Natl. Acad. Sci. USA.

[35] Maddison D.C., Mattedi F., Vagnoni A., Smith G.A. (2023). Clonal Imaging of Mitochondria in the Dissected Fly Wing. Cold Spring Harb. Protoc..

[36] Bischof J., Björklund M., Furger E., Schertel C., Taipale J., Basler K. (2013). A versatile platform for creating a comprehensive UAS-ORFeome library in Drosophila. Development.

[37] Stowers R.S., Megeath L.J., Górska-Andrzejak J., Meinertzhagen I.A., Schwarz T.L. (2002). Axonal Transport of Mitochondria to Synapses Depends on Milton, a Novel Drosophila Protein. Neuron.

[38] Glater E.E., Megeath L.J., Stowers R.S., Schwarz T.L. (2006). Axonal transport of mitochondria requires milton to recruit kinesin heavy chain and is light chain independent. J. Cell Biol..

[39] Russo G.J., Louie K., Wellington A., Macleod G.T., Hu F., Panchumarthi S., Zinsmaier K.E. (2009). Drosophila Miro is required for both anterograde and retrograde axonal mitochondrial transport. J. Neurosci..

[40] Mattedi F., Maddison D.C., Smith G.A., Vagnoni A. (2023). Live Imaging of Mitochondria in the Intact Fly Wing. Cold Spring Harb. Protoc..

[41] Storrie B., Micaroni M., Morgan G.P., Jones N., Kamykowski J.A., Wilkins N., Pan T.H., Marsh B.J. (2012). Electron tomography reveals Rab6 is essential to the trafficking of trans-Golgi clathrin and COPI-coated vesicles and the maintenance of Golgi cisternal number. Traffic.

[42] White J., Johannes L., Mallard F., Girod A., Grill S., Reinsch S., Keller P., Tzschaschel B., Echard A., Goud B., Stelzer E.H. (1999). Rab6 coordinates a novel Golgi to ER retrograde transport pathway in live cells. J. Cell Biol..

[43] Tan D., Cai Y., Wang J., Zhang J., Menon S., Chou H.-T., Ferro-Novick S., Reinisch K.M., Walz T. (2013). The EM structure of the TRAPPIII complex leads to the identification of a requirement for COPII vesicles on the macroautophagy pathway. Proc. Natl. Acad. Sci. USA.

[44] Ackema K.B., Prescianotto-Baschong C., Hench J., Wang S.C., Chia Z.H., Mergentaler H., Bard F., Frank S., Spang A. (2016). Sar1, a Novel Regulator of ER-Mitochondrial Contact Sites. PLoS One.

[45] Peter C.J., Evans M., Thayanithy V., Taniguchi-Ishigaki N., Bach I., Kolpak A., Bassell G.J., Rossoll W., Lorson C.L., Bao Z.-Z., Androphy E.J. (2011). The COPI vesicle complex binds and moves with survival motor neuron within axons. Hum. Mol. Genet..

[46] Baker N., Patel J., Khacho M. (2019). Linking mitochondrial dynamics, cristae remodeling and supercomplex formation: How mitochondrial structure can regulate bioenergetics. Mitochondrion.

[47] Zick M., Rabl R., Reichert A.S. (2009). Cristae formation—linking ultrastructure and function of mitochondria. Biochim. Biophys. Acta.

[48] Claerhout S., Dutta B., Bossuyt W., Zhang F., Nguyen-Charles C., Dennison J.B., Yu Q., Yu S., Balázsi G., Lu Y., Mills G.B. (2012). Abortive Autophagy Induces Endoplasmic Reticulum Stress and Cell Death in Cancer Cells. PLoS One.

[49] Watkin L.B., Jessen B., Wiszniewski W., Vece T.J., Jan M., Sha Y., Thamsen M., Santos-Cortez R.L.P., Lee K., Gambin T. (2015). COPA mutations impair ER-Golgi transport and cause hereditary autoimmune-mediated lung disease and arthritis. Nat. Genet..

[50] Thiel K., Heier C., Haberl V., Thul P.J., Oberer M., Lass A., Jäckle H., Beller M. (2013). The evolutionary conserved protein CG9186 is associated with lipid droplets, required for their positioning and for fat storage. J. Cell Sci..

[51] Wood J.G., McLaughlin B.J., Barber R.P. (1974). THE VISUALIZATION OF CONCANAVALIN A BINDING SITES IN PURKINJE CELL SOMATA AND DENDRITES OF RAT CEREBELLUM. J. Cell Biol..

[52] Oliva M.K., Pérez-Moreno J.J., O’Shaughnessy J., Wardill T.J., O’Kane C.J. (2020). Endoplasmic Reticulum Lumenal Indicators in Drosophila Reveal Effects of HSP-Related Mutations on Endoplasmic Reticulum Calcium Dynamics. Front. Neurosci..

[53] Konno T., Parutto P., Bailey D.M.D., Davì V., Crapart C., Awadelkareem M.A., Hockings C., Brown A., Xiang K.M., Agrawal A. (2021).

[54] Ma J.H., Shen S., Wang J.J., He Z., Poon A., Li J., Qu J., Zhang S.X. (2017). Comparative Proteomic Analysis of the Mitochondria-associated ER Membrane (MAM) in a Long-term Type 2 Diabetic Rodent Model. Sci. Rep..

[55] Cieri D., Vicario M., Giacomello M., Vallese F., Filadi R., Wagner T., Pozzan T., Pizzo P., Scorrano L., Brini M., Calì T. (2018). SPLICS: a split green fluorescent protein-based contact site sensor for narrow and wide heterotypic organelle juxtaposition. Cell Death Differ..

[56] Hewitt V.L., Miller-Fleming L., Andreazza S., Mattedi F., Prudent J., Polleux F., Vagnoni A., Whitworth A.J. (2020). Decreasing pdzd8-mediated mitochondrial-ER contacts in neurons improves fitness by increasing mitophagy. bioRxiv.

[57] Gunter T.E., Buntinas L., Sparagna G., Eliseev R., Gunter K. (2000). Mitochondrial calcium transport: mechanisms and functions. Cell Calcium.

[58] Evans C.S., Holzbaur E.L.F. (2020). Quality Control in Neurons: Mitophagy and Other Selective Autophagy Mechanisms. J. Mol. Biol..

[59] McWilliams T.G., Prescott A.R., Allen G.F.G., Tamjar J., Munson M.J., Thomson C., Muqit M.M.K., Ganley I.G. (2016). *mito* -QC illuminates mitophagy and mitochondrial architecture in vivo. J. Cell Biol..

[60] Lee J.J., Sanchez-Martinez A., Martinez Zarate A., Benincá C., Mayor U., Clague M.J., Whitworth A.J. (2018). Basal mitophagy is widespread in Drosophila but minimally affected by loss of Pink1 or parkin. J. Cell Biol..

[61] Sung H., Tandarich L.C., Nguyen K., Hollenbeck P.J. (2016). Compartmentalized Regulation of Parkin-Mediated Mitochondrial Quality Control in the Drosophila Nervous System In Vivo. J. Neurosci..

[62] Cao X., Wang H., Wang Z., Wang Q., Zhang S., Deng Y., Fang Y. (2017). *In vivo* imaging reveals mitophagy independence in the maintenance of axonal mitochondria during normal aging. Aging Cell.

[63] Csordás G., Renken C., Várnai P., Walter L., Weaver D., Buttle K.F., Balla T., Mannella C.A., Hajnóczky G. (2006). Structural and functional features and significance of the physical linkage between ER and mitochondria. J. Cell Biol..

[64] Garrido-Maraver J., Loh S.H.Y., Martins L.M. (2020). Forcing contacts between mitochondria and the endoplasmic reticulum extends lifespan in a Drosophila model of Alzheimer’s disease. Biol. Open.

[65] Xu L., Wang X., Zhou J., Qiu Y., Shang W., Liu J.-P., Wang L., Tong C. (2020). Miga-mediated endoplasmic reticulum–mitochondria contact sites regulate neuronal homeostasis. Elife.

[66] Hübner C.A., Kurth I. (2014). Membrane-shaping disorders: a common pathway in axon degeneration. Brain.

[67] Zhang B., Yu Q., Huo D., Li J., Liang C., Li H., Yi X., Xiao C., Zhang D., Li M. (2018). Arf1 regulates the ER-mitochondria encounter structure (ERMES) in a reactive oxygen species-dependent manner. FEBS J..

[68] Iwasawa R., Mahul-Mellier A.-L., Datler C., Pazarentzos E., Grimm S. (2011). Fis1 and Bap31 bridge the mitochondria-ER interface to establish a platform for apoptosis induction. EMBO J..

[69] Todd A.G., Lin H., Ebert A.D., Liu Y., Androphy E.J. (2013). COPI transport complexes bind to specific RNAs in neuronal cells. Hum. Mol. Genet..

[70] Dalla Costa I., Buchanan C.N., Zdradzinski M.D., Sahoo P.K., Smith T.P., Thames E., Kar A.N., Twiss J.L. (2021). The functional organization of axonal mRNA transport and translation. Nat. Rev. Neurosci..

[71] Pease-Raissi S.E., Pazyra-Murphy M.F., Li Y., Wachter F., Fukuda Y., Fenstermacher S.J., Barclay L.A., Bird G.H., Walensky L.D., Segal R.A. (2017). Paclitaxel Reduces Axonal Bclw to Initiate IP3R1-Dependent Axon Degeneration. Neuron.

[72] Harbauer A.B., Hees J.T., Wanderoy S., Segura I., Gibbs W., Cheng Y., Ordonez M., Cai Z., Cartoni R., Ashrafi G. (2022). Neuronal mitochondria transport Pink1 mRNA via synaptojanin 2 to support local mitophagy. Neuron.

[73] Casellas-Díaz S., Larramona-Arcas R., Riqué-Pujol G., Tena-Morraja P., Müller-Sánchez C., Segarra-Mondejar M., Gavaldà-Navarro A., Villarroya F., Reina M., Martínez-Estrada O.M., Soriano F.X. (2021). Mfn2 localization in the ER is necessary for its bioenergetic function and neuritic development. EMBO Rep..

[74] Costello J.L., Castro I.G., Hacker C., Schrader T.A., Metz J., Zeuschner D., Azadi A.S., Godinho L.F., Costina V., Findeisen P. (2017). ACBD5 and VAPB mediate membrane associations between peroxisomes and the ER. J. Cell Biol..

[75] Obara C.J., Nixon-Abell J., Moore A.S., Riccio F., Hoffman D.P., Shtengel G., Xu C.S., Schaefer K., Pasolli H.A., Masson J.-B. (2022). Motion of single molecular tethers reveals dynamic subdomains at ER-mitochondria contact sites. bioRxiv.

[76] Yamanaka T., Nishiyama R., Shimogori T., Nukina N. (2020). Proteomics-Based Approach Identifies Altered ER Domain Properties by ALS-Linked VAPB Mutation. Sci. Rep..

[77] Soussan L., Burakov D., Daniels M.P., Toister-Achituv M., Porat A., Yarden Y., Elazar Z. (1999). Erg30, a Vap-33–Related Protein, Functions in Protein Transport Mediated by Copi Vesicles. J. Cell Biol..

[78] Kamemura K., Chen C.A., Okumura M., Miura M., Chihara T. (2021). Amyotrophic lateral sclerosis-associated Vap33 is required for maintaining neuronal dendrite morphology and organelle distribution in *Drosophila*. Gene Cell..

[79] Hurd D.D., Saxton W.M. (1996). Kinesin Mutations Cause Motor Neuron Disease Phenotypes by Disrupting Fast Axonal Transport in Drosophila. Genetics.

[80] Woźniak M.J., Bola B., Brownhill K., Yang Y.-C., Levakova V., Allan V.J. (2009). Role of kinesin-1 and cytoplasmic dynein in endoplasmic reticulum movement in VERO cells. J. Cell Sci..

[81] Friedman J.R., Webster B.M., Mastronarde D.N., Verhey K.J., Voeltz G.K. (2010). ER sliding dynamics and ER–mitochondrial contacts occur on acetylated microtubules. J. Cell Biol..

[82] Zamponi E., Meehl J.B., Voeltz G.K. (2022). The ER ladder is a unique morphological feature of developing mammalian axons. Dev. Cell.

[83] Bettayeb K., Hooli B.V., Parrado A.R., Randolph L., Varotsis D., Aryal S., Gresack J., Tanzi R.E., Greengard P., Flajolet M. (2016). Relevance of the COPI complex for Alzheimer’s disease progression in vivo. Proc. Natl. Acad. Sci. USA.

[84] Astroski J.W., Akporyoe L.K., Androphy E.J., Custer S.K. (2021). Mutations in the COPI coatomer subunit α-COP induce release of Aβ-42 and amyloid precursor protein intracellular domain and increase tau oligomerization and release. Neurobiol. Aging.

[85] Stevenson-Hoare J., Heslegrave A., Leonenko G., Fathalla D., Bellou E., Luckcuck L., Marshall R., Sims R., Morgan B.P., Hardy J. (2023). Plasma biomarkers and genetics in the diagnosis and prediction of Alzheimer’s disease. Brain.

[86] Lau D.H.W., Paillusson S., Hartopp N., Rupawala H., Mórotz G.M., Gomez-Suaga P., Greig J., Troakes C., Noble W., Miller C.C.J. (2020). Disruption of endoplasmic reticulum-mitochondria tethering proteins in post-mortem Alzheimer’s disease brain. Neurobiol. Dis..

[87] Area-Gomez E., de Groof A., Bonilla E., Montesinos J., Tanji K., Boldogh I., Pon L., Schon E.A. (2018). A key role for MAM in mediating mitochondrial dysfunction in Alzheimer disease. Cell Death Dis..

[88] Hewitt V.L., Miller-Fleming L., Twyning M.J., Andreazza S., Mattedi F., Prudent J., Polleux F., Vagnoni A., Whitworth A.J. (2022). Decreasing pdzd8-mediated mito–ER contacts improves organismal fitness and mitigates Aβ _42_ toxicity. Life Sci. Alliance.

[89] Custer S.K., Todd A.G., Singh N.N., Androphy E.J. (2013). Dilysine motifs in exon 2b of SMN protein mediate binding to the COPI vesicle protein α-COP and neurite outgrowth in a cell culture model of spinal muscular atrophy. Hum. Mol. Genet..

[90] Custer S.K., Foster J.N., Astroski J.W., Androphy E.J. (2019). Abnormal Golgi morphology and decreased COPI function in cells with low levels of SMN. Brain Res..

[91] Neve A., Trüb J., Saxena S., Schümperli D. (2016). Central and peripheral defects in motor units of the diaphragm of spinal muscular atrophy mice. Mol. Cell. Neurosci..

[92] Miller N., Shi H., Zelikovich A.S., Ma Y.-C. (2016). Motor neuron mitochondrial dysfunction in spinal muscular atrophy. Hum. Mol. Genet..

[93] Lotti F., Imlach W.L., Saieva L., Beck E.S., Hao L.T., Li D.K., Jiao W., Mentis G.Z., Beattie C.E., McCabe B.D., Pellizzoni L. (2012). An SMN-dependent U12 splicing event essential for motor circuit function. Cell.

[94] Van Alstyne M., Lotti F., Dal Mas A., Area-Gomez E., Pellizzoni L. (2018). Stasimon/Tmem41b localizes to mitochondria-associated ER membranes and is essential for mouse embryonic development. Biochem. Biophys. Res. Commun..

[95] Kemal S., Richardson H.S., Dyne E.D., Fu M.M. (2022). ER and Golgi trafficking in axons, dendrites, and glial processes. Curr. Opin. Cell Biol..

[96] Davidson S.M., Duchen M.R., Palmeira C.M., Moreno A.J. (2018). Mitochondrial Bioenergetics Methods in Molecular Biology.

[97] Yu Y.V., Li Z., Rizzo N.P. (2011). Targeting the motor regulator Klar to lipid droplets. BMC Cell Biol.

[98] Chang Y.Y., Neufeld T.P. (2009). An Atg1/Atg13 complex with multiple roles in TOR-mediated autophagy regulation. Mol Biol Cell..

[99] Lu M., Van Tartwijk F.W., Lin J.Q., Nijenhuis W., Parutto P., Fantham M., Christensen C.N., Avezov E., Holt C.E., Tunnacliffe A. (2020). The structure and global distribution of the endoplasmic reticulum network are actively regulated by lysosomes. Sci. Adv..

