# Supplementary material for: COPI-regulated mitochondria-ER contact site formation maintains axonal integrity
Source: Cell Rep. 2023 Jul 26;42(8):112883. doi: 10.1016/j.celrep.2023.112883 (PMC10840514; doi:10.1016/j.celrep.2023.112883)
Supplement: Document S1. Figures S1–S6 [file mmc1.pdf]

Cell Reports, Volume *42*

Supplemental information

COPI-regulated mitochondria-ER contact site formation maintains axonal integrity

Daniel C. Maddison, Bilal Malik, Leonardo Amadio, Dana M. Bis-Brewer, Stephan Züchner, Owen M. Peters, and Gaynor A. Smith

Figure S1

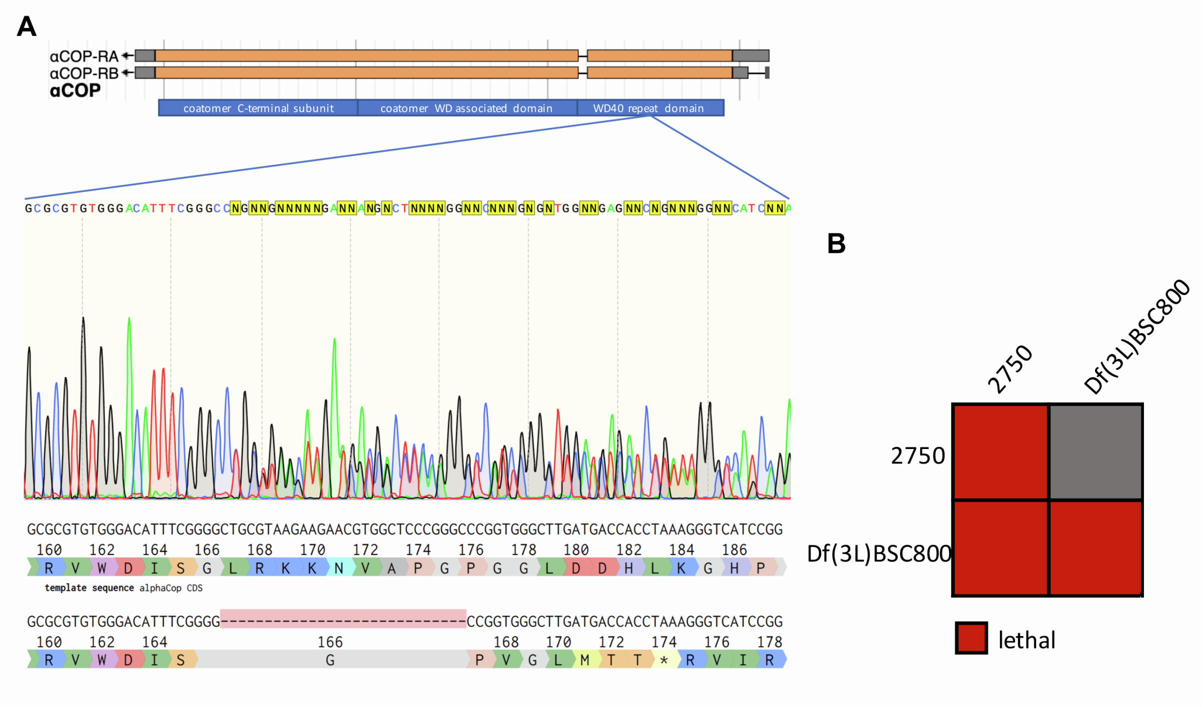

Figure S1. Mutation 2750 is a null mutant of *αCop*. A) Sanger sequencing of a heterozygous l(3)2750 mutant, which features a 28 bp deletion. **B)** Complementation scheme of mutant - crossed to a deficiency strain a deletion including the *αCop* locus indicates mutant l(3)2750 is a null.

Figure S2

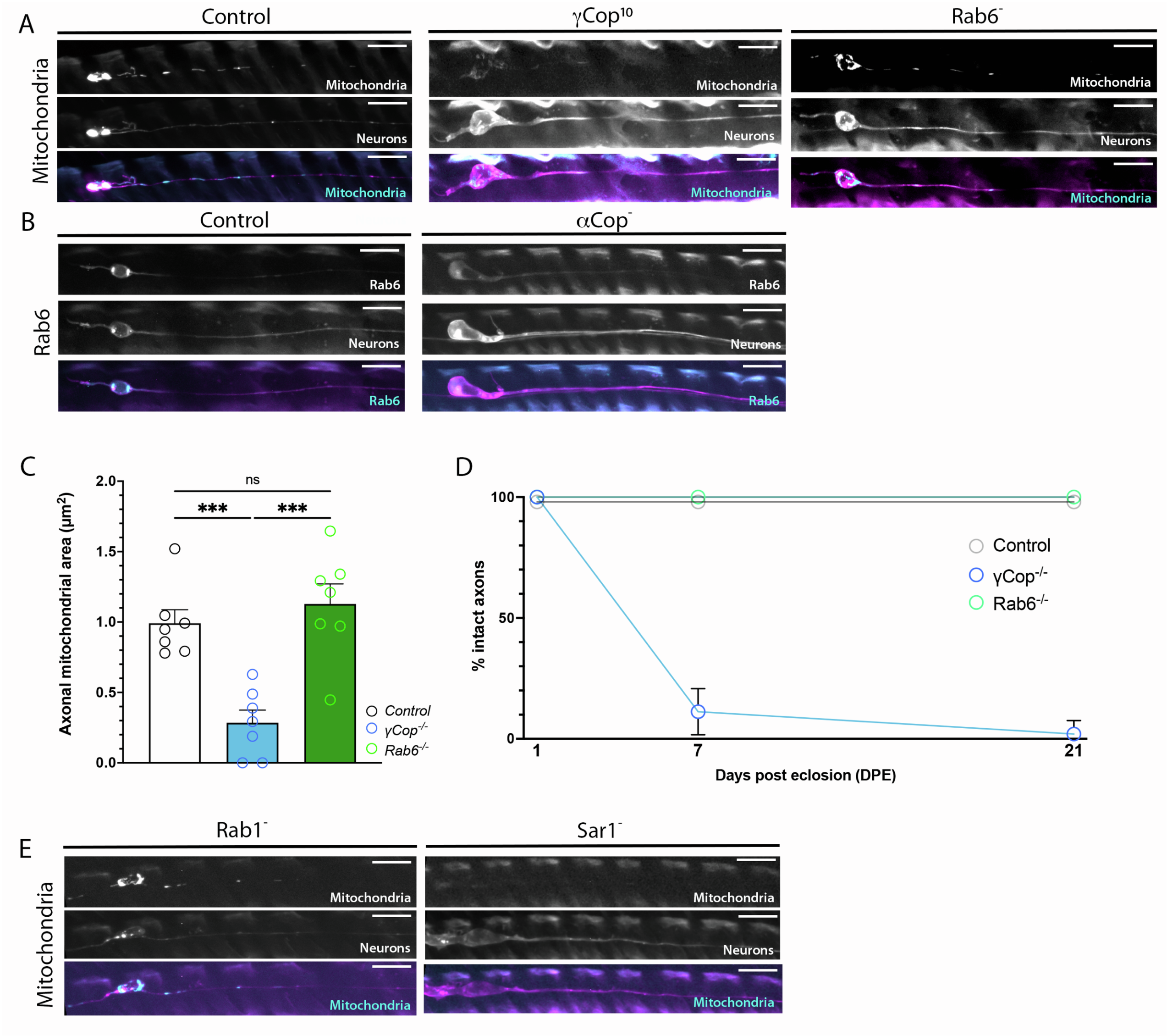

Figure S2. Mitochondrial morphology is COPI complex-dependent and Rab6 / Rab1 independent. A) Mito::GFP labelled mitochondria myr::tdTomato labelled neuronal membrane in control, *γCop*^{10} and *Rab6*$^{-}$ mutant *Drosophila* wing neurons at 1, 7 & 21 DPE. **B)** Rab6 positive vesicles labelled with *UAS-Rab6::GFP* are reduced in *αCop*$^{-}$ and *Rab6*$^{-}$ mutant *Drosophila* wing neurons at 1 DPE. **C)** Median mitochondrial area was assessed in axons *γCop*^{10} and *Rab6*$^{-}$ mutant *Drosophila* wing neurons, and was significantly reduced by γCop depletion compared to control. **D)** Percentage intact axons were reduced in γCop ablated conditions. **E)** *Rab1*$^{-}$ clones did not phenocopy *αCop*$^{-}$, however *Sar1*$^{-}$ caused a dramatic reduction in mitochondrial mass. Significance was assessed through one-way ANOVA with FDR correction. Graphs are expressed as Mean ± SD, Each data point represents mean value for an

individual fly, calculated across 5-10 clones from both wings. Graphs are annotated as: ns = not significant and *** $P < 0.001$. Scale bars = 10 μm.

Figure S3.

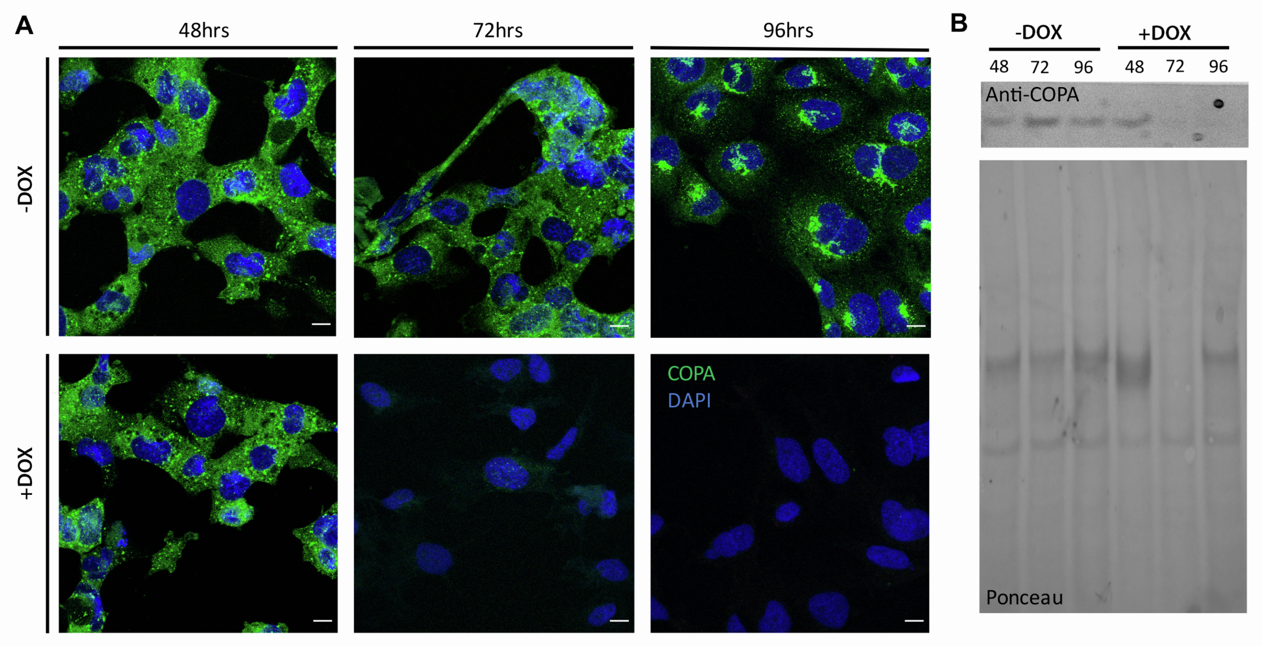

Figure S3. Inducible COPA-targeting shRNA reduces COPA levels 72hrs post-induction in SH-SY5Y neuroblastoma cells. A) Control and COPA KD SH-SY5Y cells at 48, 72 and 96 hrs post-dox treatment labelled with anti-COPA and DAPI-stained nuclei. **B)** Immunoblot of total protein extract of control and COPA KD SH-SY5Y cells at 48, 72 and 96 hrs post-dox treatment. Total protein was visualized by Ponceau stain. COPA was significantly depleted by 72hrs after Doxycycline treatment. Scale bars = 10 μm.

Figure S4.

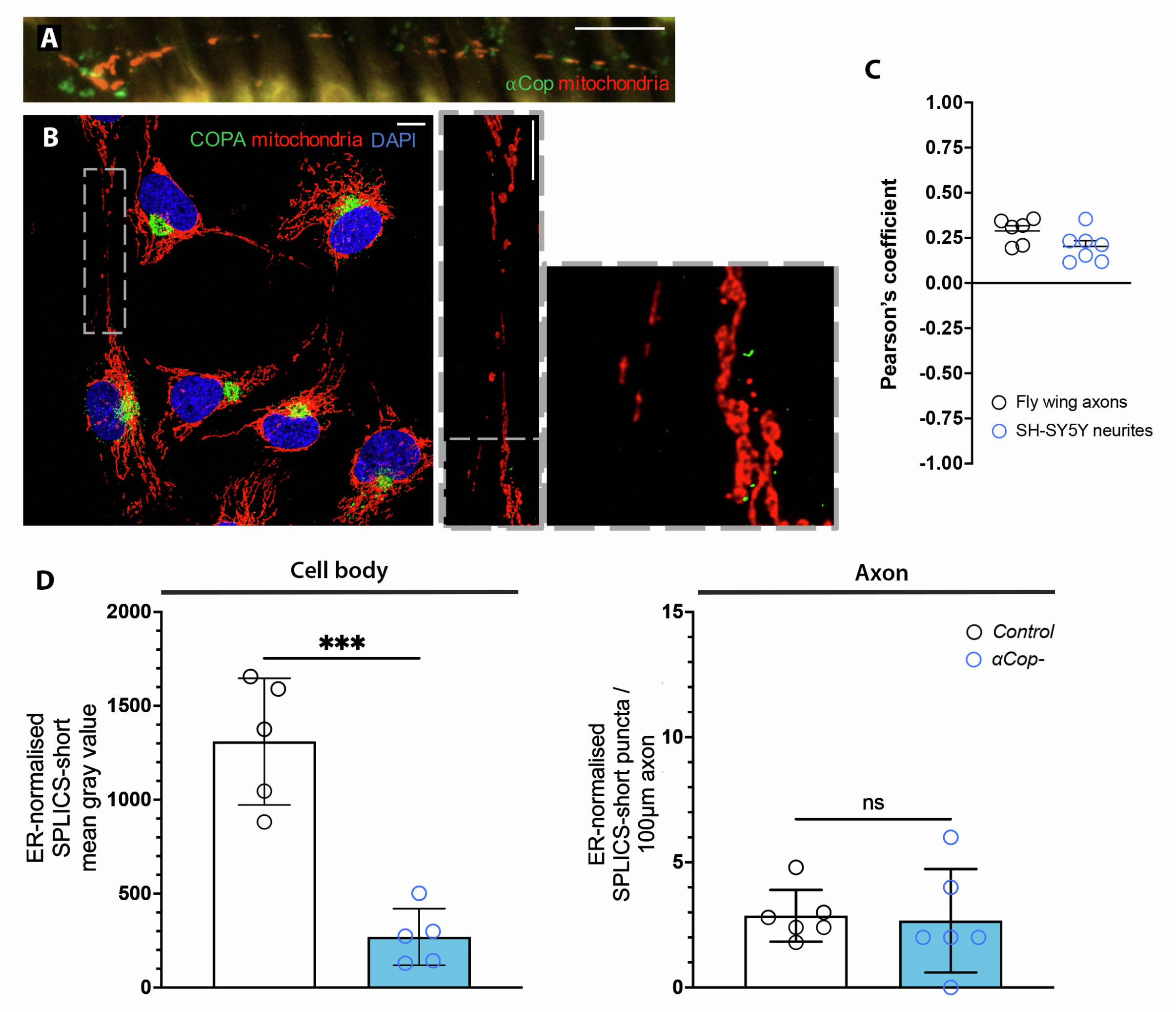

Figure S4. αCop/COPA partially co-localizes with mitochondria and *αCop*-induced MERCS depletion in axons correlates with loss of ER structure. A) mito::tdTomato and aCop::GFP labelled in *Drosophila* neurons. **B)** SH-SY5Y cells labelled with anti-TOMM20 for outer-mitochondrial membrane, anti-COPA and DAPI-stained nuclei. **C)** Pearson's coefficient analysis of colocalization between *αCop* and mitochondria showed a partial overlap *in vivo* and *in vitro*. Scale bars = 10 µM. **D)** SPLICS-short mean gray value in cell body and puncta in axons, normalized to fluorescence levels of Sturkopf::GFP in Control vs αCop- neurons. Individual data points represent mean values from individual flies, calculated across 5-10 clones from both wings. Graphs were expressed in Mean ± SD and annotated as: ns – not significant, *** $P < 0.001$.

Figure S5.

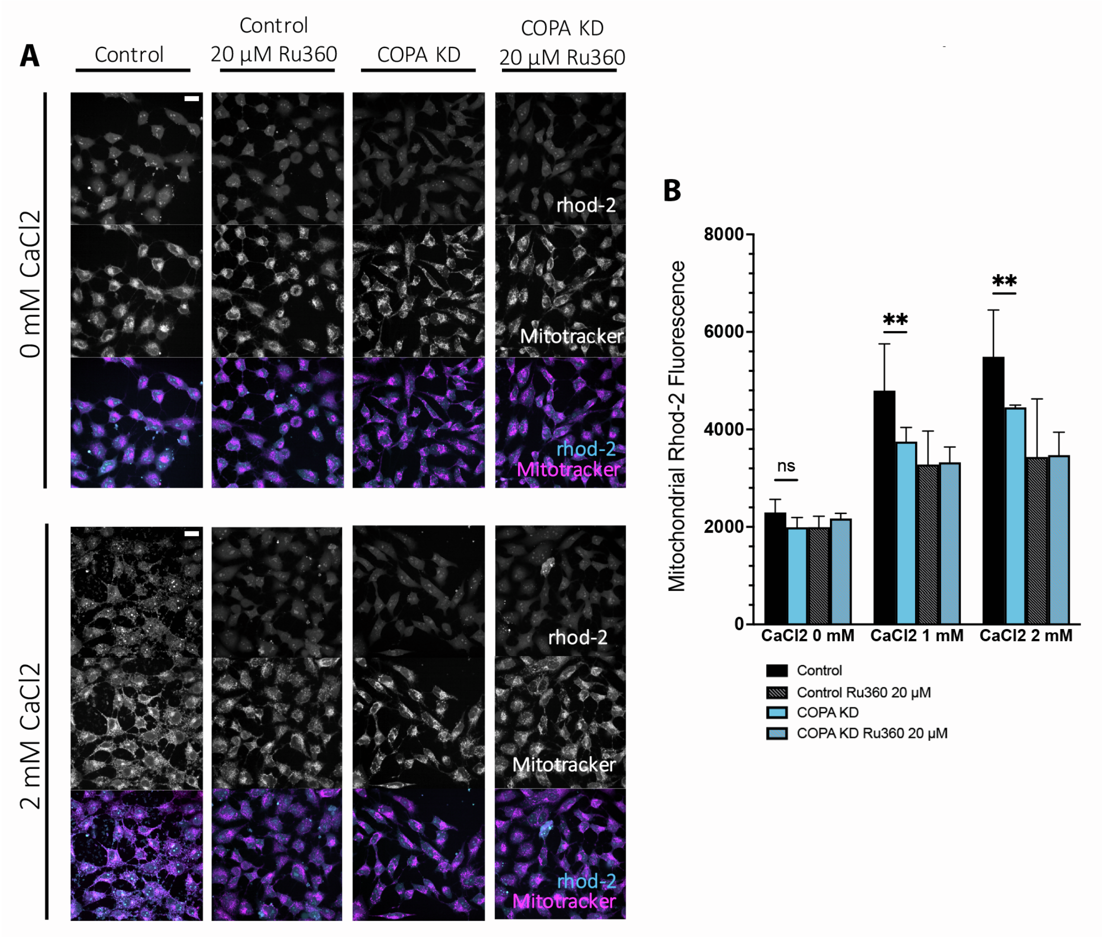

Figure S5. Mitochondrial Ca^{2+} uptake is perturbed in SH-SY5Y cells depleted of COPA. A) Mitochondrial Ca^{2+} was visualized using the Rhod-2 fluorescent dye in SH-SY5Y cells co-labeled with Mitotracker. Under CaCl2 inhibited conditions no mitochondrial Ca^{2+} uptake was observed following COPA knockdown stimulated by Ru360 treatment (top). However, in 2 mM CaCl2 conditions mitochondria in COPA knockdown cells failed to uptake Ca^{2+} in contrast to control cells or cells without Ru360 treatment (bottom). **B)** Quantification shows a significant increase in mitochondrial Rhod-2 fluorescence at both 1 mM and 2 mM CaCl2 conditions following COPA knock down compared to control. Data was analysed by two-way ANOVA with FDR correction. Graphs are expressed as Mean ± SD (of 6 wells per condition) and annotated with: ** $P < 0.01$. Scale bars = 10 µm.

Figure S6.

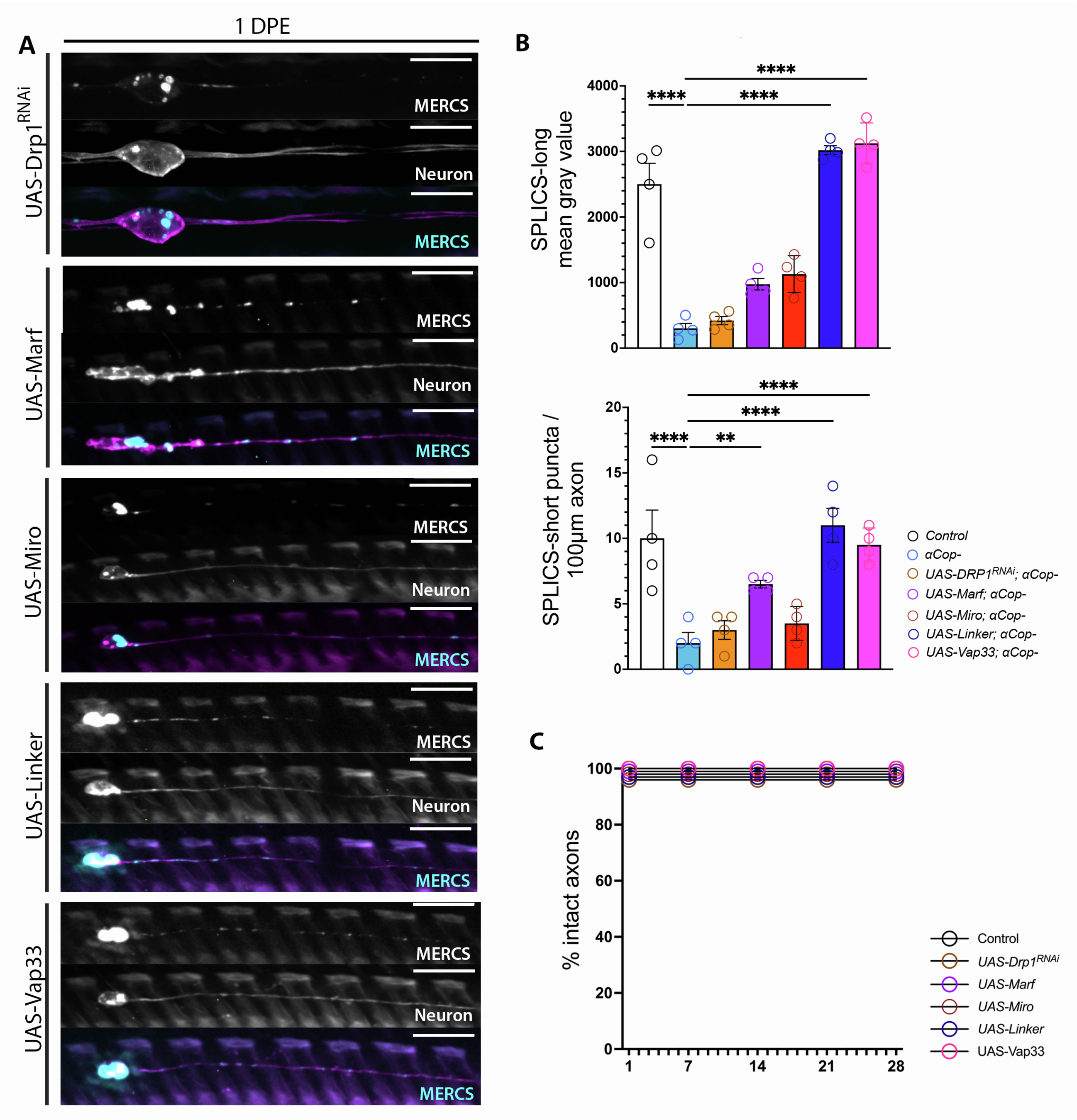

Figure S6. Enhancing mitochondrial dynamics and membrane tethering increase MERCS formation in αCop depleted neurons. A) SPLICS reporters was used to visualize MERCS in *αCop⁻* mutant *Drosophila* wing neurons at 1DPE upon: Drp1 knockdown using *UAS-Drp1^{RNAi}*, Marf overexpression using *UAS-Marf*, Miro overexpression using *UAS-Miro*, ER and mitochondria tethering using *UAS-Linker* and *UAS-Vap33* overexpression. *UAS-Sturkopf::GFP* was used to mark neuronal ER. **B)**

Quantification shows that short and long MERCS are restored to wild type levels in *αCop*$^{-}$ mutant *Drosophila* axons following artificial tether using Linker or by Vap33 overexpression. Overexpression of Marf partially restored axonal MERCS **C)** At 1 DPE neither altered mitochondrial dynamics or enhanced MERCS formation impacted on wild type neuron survival. Data was analysed by one-way or two-way ANOVA with FDR correction. Each data point represents mean value for an individual fly, calculated across 5-10 clones from both wings. Graphs are expressed as Mean ± SD and annotated with: ** $P < 0.01$, **** $P < 0.0001$. Scale bars = 10 µm.
